# Combining machine learning and quantum chemical calculations for high-throughput virtual screening of thermally activated delayed fluorescence molecular materials: the impact of selection strategy and structural mutations†

**DOI:** 10.1039/d2ra05643g

**Published:** 2022-10-31

**Authors:** Chunyun Tu, Weijiang Huang, Sheng Liang, Kui Wang, Qin Tian, Wei Yan

**Affiliations:** School of Chemistry and Materials Engineering, Guiyang University Guiyang 550005 P. R. China lrasyw@163.com +86-180-9605-0905; School of Mathematics and Information Science, Guiyang University Guiyang 550005 P. R. China

## Abstract

In view of the theoretical importance and huge application potential of Thermally Activated Delayed Fluorescence (TADF) materials, it is of great significance to conduct High-Throughput Virtual Screening (HTVS) on compound libraries to find TADF candidate molecules. This research focuses on the computational design of pure organic TADF molecules. By combining machine learning and quantum chemical calculations, using cheminformatics tools, and introducing the concept of selection and mutation from evolutionary theory, we have designed a computational program for HTVS of TADF molecular materials, especially the impact of selection strategy and structural mutations on the results of HTVS was explored. An initial compound library (size = 10^3^) constructed by enumeration of typical donors and acceptors was used to evolve by successively applying selection and 10 different structural mutations. And a group fingerprint similarity (Δ_MSPR_) index was proposed to account for the similarity between two compound libraries with comparable sizes. Based on the computed data, we have found that the mix of selection and mutations into the evolution map does have great impact on the HTVS results: (a) except the fast mutation **Sub2**, all the rest of the mutations can effectively concentrate ‘good’ molecules in a compound library, and hence give large material abundance (typically >0.8) for high mutation generations (*n*_g_ ≥ 6). (b) The mean energy gap can exhibit a fast convergent trend toward very low values, hence the studied mutations (except **Sub2**) can cooperate very well with the studied DA substrates to generate optimal molecules, and the group fingerprint similarity can retain high enough values for large *n*_g_, which can be associated with the apparent convergence in molecular skeletons as *n*_g_ increases. (c) The distribution of skeleton frequencies for a specific mutation is generally uneven with one dominant skeleton. The overall numbers of common and generic cores for all mutations are 11 and 7 as *n*_g_ = 9. Hence, in a sense, the ‘optimal’ skeletons seem unique and useful in realizing low energy gaps. With these observations and the development of related HTVS software, we expect to provide insight and tools to the research community of HTVS of molecular (TADF) materials.

## Abstract 2

The combination of machine learning, quantum chemical computation with evolutionary theory (selection and mutation) makes high-throughput virtual screening of organic thermally activated delayed fluorescence molecular materials simple.

## Graphical abstract

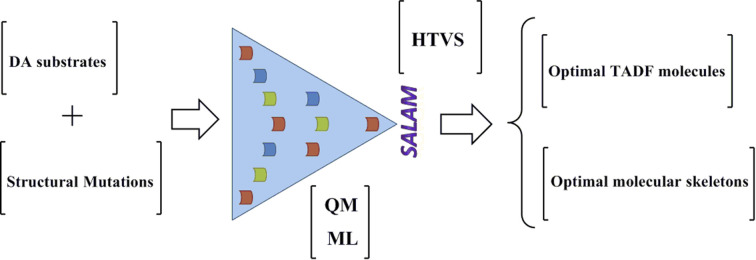

## 1 Introduction

Since Tang and VanSlyke's first big breakthrough in 1987,^1^ organic light-emitting diodes (OLEDs) have been profoundly improved in materials, device structures, and luminous efficiency.^2,3^ In recent years, OLEDs have been widely used in the manufacture of display devices (such as TV screens, computer monitors, smart phone screens, flexible display panels, *etc.*), and are considered to have great potential in the field of lighting.^4^ A modern OLED typically has a three-layer architecture, [anode|hole transport layer|light-emitting layer|electron transport layer|cathode]. The light-emitting material is dispersed in the light-emitting layer by doping or non-doping manner, and emits light in response to the current generated by the potential applied across the electrodes, which is so-called electroluminescence.^4^

So far, the luminescent materials as core OLED materials have undergone profound improvements, starting from the first generation of fluorescent materials (*e.g.*, aluminum octahydroxyquinoline), through the second generation of phosphorescent materials represented by heavy transition noble metal organic complexes (*e.g.*, bipyridine complexes of Ir(iii)) until the third generation of TADF materials (*e.g.*, organic donor–π-bridge–acceptor molecules).

**Fig. 1:**
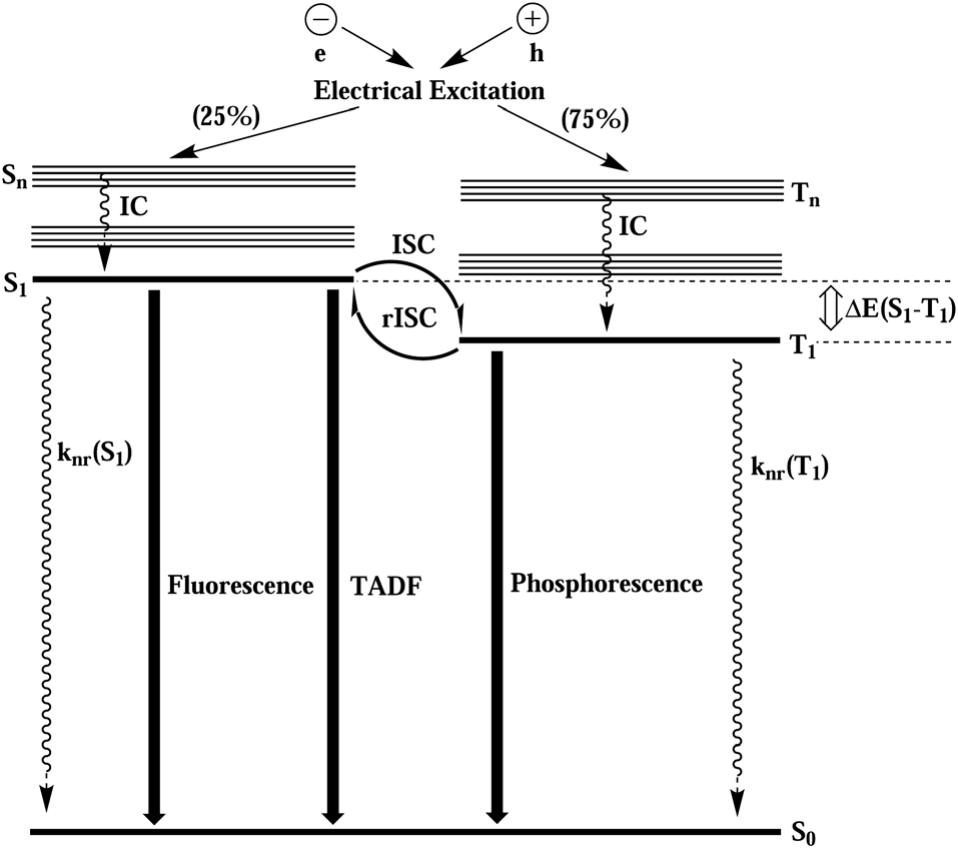
Schematic diagram of the electroluminescence process of thermally activated delayed fluorescent materials.

Upon electric excitation, TADF materials (compounds characterized by very low first excited singlet-triplet energy gaps (Δ*E*_ST_)) get thermally activated to induce efficient reverse intersystem crossing (rISC) where the triplet excitons get converted into singlet excitons, so as to emit light dominantly from the emissive singlet excited state. In Fig. 1, the electroluminescence process of TADF material is schematically shown. Compared with noble metal–organic complex phosphorescent materials, TADF materials have the advantages of larger material space, low price, easy preparation and synthesis, easy fabrication of flexible screens, and more stable blue light emission. Therefore, in the last decade, as the most promising electroluminescent material for modern OLEDs, they have been experimentally,^2,5–9^, theoretically^10–23^ and theory-experiment jointly^15,24,25^ studied in depth.

Basically, there are two classes of TADF materials that have been carefully explored.^4^ The first type is pure organic D–A or D–π–A systems whose electron donor (D) or acceptor (A) are mainly constructed by nitrogen-containing aromatic heterocycles. The lowest excitation states typically possess significant intramolecular charge transfer (CT) transition character. After reasonable design and optimization, the external quantum efficiency (EQE) of OLED devices based on such TADF materials can even be as high as 30%. From the perspective of structural characteristics, the best luminous efficiency usually corresponds to the twisted D–A (or D–π–A) compounds due to enough steric hindrance between the donor and acceptor parts. Another type is transition metal (Cu(i), Ag(i), Zn(ii), *etc.*) complexes with electronic configuration of d^10^, and their lowest excited states usually have significant metal–ligand Charge transfer (MLCT) transition character. The saturated d^10^ electronic configuration of the central metal is very beneficial to reduce the possible quenching of the dπ–dπ* transitions in the complex and achieve deep blue emission.

The experimental breakthroughs came mainly from Adachi and collaborators, who focused on designing organic molecules with D–π–A (and other) frameworks, and tuning the frameworks to achieve a small enough Δ*E*_ST_ while maintaining a suitable fluorescence radiation rate, so that efficient TADF becomes possible. Recently developed blue TADF OLED devices have an EQE approaching 37%, which is rather impressive considering the EQE of Tang and VanSlyke's 1987 version of fluorescent OLEDs is about 1%.^1^

In a review on molecular design patterns of organic TADF materials,^3^ Im *et al.* suggested that high-efficiency TADF materials should have at least a small Δ*E*_ST_ and a high photoluminescence quantum yield (PLQY). Δ*E*_ST_ is associated with upconverting triplet excitons to singlet excitons, while PLQY is closely related to the radiative transition probability. To obtain a small Δ*E*_ST_, a strong donor/acceptor should be used and the molecular backbone should be twisted. The acquisition of high PLQY should have: a phenyl bridge as a connecting unit, delocalized and dispersed highest occupied molecular orbital (HOMO), and a double luminescent core. These strategies will undoubtedly provide useful guidance for further molecular design of TADF materials.

Contemporary electronic structure theory methods (*e.g.*, density functional theory, DFT) have been able to predict the optoelectronic properties of molecules (or materials) with relatively high accuracy.^26,27^ Theoretical research is playing an increasingly important role in the in-depth understanding of the structure–property relationship and luminescence mechanism of TADF materials, and has a significant impact on the molecular design of such materials. As pointed out by Olivier and collaborators, theoretical research on this type of materials requires careful consideration.^19^ Designing new molecules with efficient TADF emission is a difficult task, as they must exhibit a strong transition between singlet and triplet states without using heavy elements to enhance spin–orbit coupling fast conversion (large *k*_rISC_). They should also show a large fluorescence rate (large *k*_F_), but at the same time a small energy difference between excited singlet and triplet states (small Δ*E*_ST_). In a feature article, Penfold *et al.* reviewed recent advances in theoretical and computational chemistry to understand TADF materials and mechanisms.^20^ For luminescence dynamics, simply assume *k*_rISC_ ≫ *k*_F_, and apply eqn (1)1
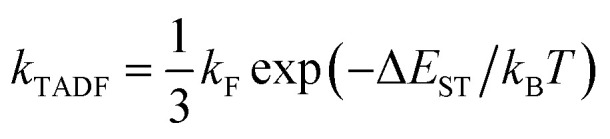
for rate estimation is considered inappropriate, and a new way to uniformly deal with the relevant quantities is needed. For electronic structure calculations, the standard time-dependent density functional method (TDDFT) may fail, in which case a tuned range-separated hybrid DFT or multi-reference configuration interaction (MRCI) method needs to be introduced. To understand the TADF mechanism, it is not enough to calculate the reverse intersystem crossover rate (*k*_rISC_) between singlet (S_1_) and triplet (T_1_) only by using first-order perturbation theory and Fermi's golden rule. Rather, the second-order perturbation theory including the spin-vibronic mechanism needs to be taken into account. In addition, conformational, regioisomerization, as well as environmental effects, are also crucial in determining the properties of TADF materials, and should therefore also be taken into account.

Commonly viewed as a branch discipline of theoretical chemistry, the rise of cheminformatics in recent years is deemed to make great impact on chemical science. With the continuous development of the theoretical system,^28–33^ additionally, the open-sourceization of many high-quality cheminformatics tools (*e.g.*, RDKit, Mordred, stk *etc.*),^34–37^ those make it possible (even for non-experts) to efficiently manage large amounts of chemical information. The efficient management of virtual molecules as well as molecular libraries *in silico* by using cheminformatics tools is crucial for large-scale computational design of (molecular) materials. On the other hand, in the field of computational design of (organic) molecules and (solid-state) materials, exploration of chemical compound space (CCS) using high-throughput virtual screening (HTVS) methods is being accepted as a routine procedure for molecular or material lead discovery. The important material categories involved include photovoltaic materials, optoelectronic materials, organic matrix flow battery materials, *etc.*^38–40^ By designing computational funnels to efficiently deploy computational programs, the HTVS approach allows researchers to make data-driven discoveries by observing trends in the data.

As one of the branches of artificial intelligence (AI), machine learning (ML) can efficiently extract hidden relationships from large amounts of complex data. With advances in algorithmic models and open-source tools (general purpose: Scikit-learn, TensorFlow, Pytorch *etc.*;^41–44^ chemistry or materials orientation: DeepChem, MLatom, MAST-ML *etc.*^45–48^), ML has profoundly changed the research paradigm of computational chemistry (or materials) science in the last decade.^49^ Classical algorithm developments and applications include: predicting molecular atomization energies;^50^ finding density functionals for model systems;^51^ improving high-level electron correlation methods, learning universal molecular force fields; predicting molecular thermochemical properties, chemical reaction active sites, molecular excited state properties, molecular crystallization behavior, *etc.*^39,52–55^ On the other hand, the establishment of open-source molecular databases has also promoted the development and calibration of models and algorithms which combine quantum chemistry with machine learning.^45,56–59^

Considering the rarity and high price of heavy metal transition metal complex phosphorescent materials, as well as the difficulty in achieving high-performance blue light emission, it is undoubtedly very attractive to design and develop stable and efficient TADF blue light materials as an alternative.^4^ A pioneering attempt at high-throughput virtual screening of organic TADF materials was first made by Aspuru-Guzik and collaborators. By utilizing machine learning and time-dependent density functional theory methods, the screening procedure is rationally set to screen thousands of promising candidate TADF molecules from a search space of 1.6 million molecules, among which the best candidate molecules can be used to prepare OLED devices. The achieved external quantum efficiencies can be as high as 22%.^15^ In another distinguished study, the same authors designed a deep neural network incorporating a variational autoencoder (VAE),^60^ by accepting hundreds of thousands of existing chemical structures to build three coupled functions: encoder, decoder and predictor.^61^ This model can convert discrete molecular representations to and from multidimensional continuous ones. Notably, the continuous representation allows the use of powerful gradient-based optimization to efficiently guide the search for optimal functional compounds.

This study focuses on the computational design of pure organic TADF molecules, by examining the effects of structural mutations as well as selection strategy on the results of high-throughput virtual screening of TADF materials, we expect to provide theoretical basis and guidance for the optimization of organic (or metallic complex type) TADF materials (lead) for larger-scale chemical space exploration in the future.

## 2 Theoretical methods

Sieving materials effectively within a large chemical compound space is as difficult as finding a needle in a haystack. Organic TADF molecules are typically electron donor–acceptor systems (DA, D–π–A, *etc.*) with N-containing heteroaromatic rings as building blocks. By introducing the concept of mutation and selection from genetic algorithm^62^ (GA) and combining machine learning algorithm with quantum chemical computations in the calculation steps, a relatively simplified calculation program is proposed. The main aim is to efficiently explore the chemical compound space to obtain organic TADF material candidates.

Structural mutations can play an important role in tuning the electronic properties of molecular systems. Suppose our starting molecule is Biphenyl with 10 aromatic C–H bonds (aC–H) in the structure, if we allow two types of simple structural mutations:

(1) The whole is replaced by an aromatic N (aN),aC–H → aN;

(2) The terminal H is substituted by a group G (G is a common simple electron donor or acceptor),aC–H → aC–G

If we further set substitution group G to be F (Fluorine group), for this molecule, we would virtually have 2^10^ mutant offspring (assuming all positions are distinguishable), and the real size would be 210 after removing the duplicates. This only considers the consequences of a single mutation. If there are more than one possible mutations at a single substitutable position, the number of combinations will expand dramatically beyond the calculable extent, given typically available computation resources owned by a computational research group. Obviously, the size of our initial molecular library G_0_ will not be 1 (typically greater than 10^3^). Therefore, designing computational funnels based on a core property (or several core properties) of a material is crucial for efficient exploration of chemical compound space. For TADF material in current case, this property was chosen to be the energy difference between the first singlet excited state and the first triplet excited state (Δ*E*_ST_).

Both single and mixed mutations have been taken into account. They are: N(slow), N(fast), F, CN, OMe, and NMe_2_; F or OMe, F or NMe_2_, CN or OMe, CN or NMe_2_. N(slow) and N(fast) denote different mutation speeds, where N(slow) restricts only one position to be substituted, and N(fast) allows at most two. These mutations are denoted symbolically as **Sub1**, **Sub2**, **Sub3**, …, **Sub10**, respectively.

**Fig. 2:**
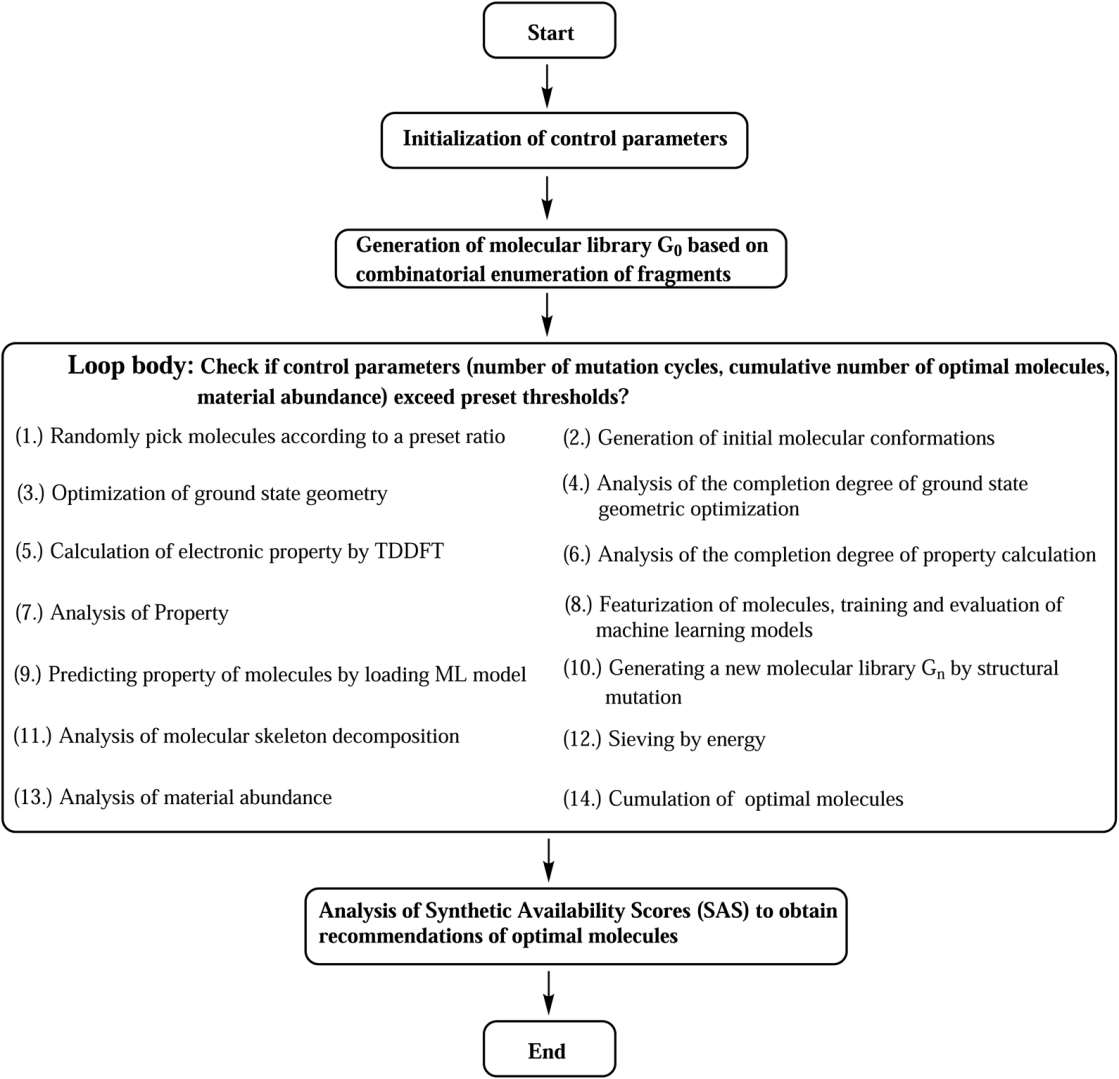
Computational road map for HTVS of TADF molecules.

The designed computational framework for high-throughput virtual screening for TADF materials in this study is schematically presented in Fig. 2. The brief process is as follows:

(a) The control parameters get initialized. The convergence criteria for the loop are set as a combination of three: number of generation of mutations (*n*_g_), number of accumulated optimal molecules (*n*_acc_opt_mols_), and material abundance (*ω*_MA_).

(b) Through rational selection of donor and acceptor fragments (30 donors and 43 acceptors, see Fig. S1 and S2 in the ESI†), under the donor–acceptor (DA) structural framework, using the open source cheminformatics package RDKit, and based on the Simplified Molecular Input Line Entry System (SMILES),^29^ an initial molecular library G_0_ (limit its size to 10^3^) was obtained by combinatorial enumeration of fragments.

(c) Starting from this library, some molecules are randomly selected (the selection ratio is set to 10%), and their initial molecular conformations are generated by the RDKit package, where the ETKGD algorithm^35^ is adopted.

(d) The core properties of the selected molecules are quickly and accurately calculated by quantum chemical calculations. The geometry optimization of ground state is performed by semi-empirical quantum chemical methond PM6-D3.^63,64^ Based on the optimized geometry, the vertical energy gap (Δ*E*_ST_) is calculated by TD-ωB97XD/6-31G(d) method.^65^

The differences between ground state geometries computed by B3LYP/6-31G(d) and PM6-D3 levels of theory is mesured by the root-mean-square deviation (RMSD) of the computed molecules for **Sub3** (*n*_g_ = 0 only). The RMSDs is calculated by the Python code rmsd with adoption of the Kabsch algorithm to align molecules.^66,67^ The distribution of frequency of RMSDs is given in Fig. S3.† The mean of the RMSDs is 0.59, and 75% of them are smaller than 0.69, which indicates the size of difference in geometries might be acceptable. Hence, the PM6-D3 method is adopt. In addition, the effect of varied ground state geometry optimization methods on the HTVS results have been briefly tested (see ESI†). Moreover, tuned range-separated hybrid functional methods (*e.g.*, LC-ω*PBE, ω*B97XD and CAM-B3LYP) are typically chosen to accurately compute the related electronic properties of TADF molecules. In this study, owing to the limit on available computational resources, the TD-ωB97XD/6-31G(d) method is chosen with the range-separation parameter not tuned, with the hope that the tuned range-separation parameters of molecules could not deviate considerably from the default values or if the deviations are considerable, they could induce the same direction changes on the distribution of the computed property of the compound library.

(e) The molecular structures get featurized by molecular fingerprint method, and are introduced into the machine learning algorithm to train and learn a model. The chosen fingerprint is the ECFP method, and the computation is assisted by the DeepChem package. And the Random Forest (RF) Regressor^68^ of the machine learning package Scikit-learn is used. (For more details, refer to the related section in the ESI.†)

(f) By using the learned ML model, we predict the property of entire molecular library so as to obtain the optimal molecules within the library.

(g) A certain proportion (10%) of the top-ranked molecules are taken out to generate a new generation of molecular libraries (named G_*n*_, *n* = 1, 2, 3, …) by means of structural mutations. Here, selection and mutation get incorporated into the computational paths.

(h) Analysis of molecular skeleton decomposition is performed to access the corresponding evolution of skeleton of molecules in library. There the Murcko Skeleton Decomposition^69^ method in the RDKit package is adopted.

(i) An energy sieve is then applied to divide the molecules into regions of different colors. Molecules with predicted vertical first excited energies (*E*_S1_) larger than 2.80 eV, between 2.50 to 2.80 eV, and smaller than 2.50 eV are partitioned into the blue, green, and red regions of colors, respectively.

(j) Compute material abundance (*ω*_MA_) and accumulate optimal molecules to get number of accumulated optimal molecules (*n*_acc_opt_mols_). The threshold for 'good' material is the predicted Δ*E*_ST_ < 0.15 eV. The material abundance is computed by eqn (2)2
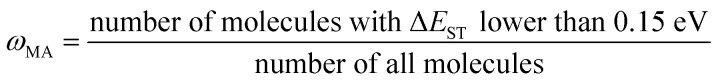


(k) The accumulated optimal molecules are finally ranked based on Synthetic Accessibility Scores (SAS) to obtain the best TADF material candidates. Low SASs imply relative ease of synthesis of molecules. Since the perpetual mutations on the molecular framework would profoundly disturb the structure, even could make the synthesis impossible, a final control of SAS is certainly necessary to sieve out bad structures from the good ones.

Repeat the above steps from (c) to (j) using the newly formed library G_*n*_ to generate a next library G_*n* + 1_, until we have reached the preset loop convergence criterion. The definition of calculation completeness for geometry optimization and property evaluation is meant to assist the automation of related calculation routines. Unavoidably, the geometry or property of some molecules (and their mutation offspring) may not converge under the chosen computational methods, therefore, only a preset completeness ratio is required to escape the steps. For geometry optimization and property calculation, the ratios are 0.80 and 0.90, respectively. Inside the loop, the interconversion of chemical files between different formats is facilitated by the Open Babel cheminformatics tool.^70^ Gratefully, the analysis of data is assisted by the Anaconda3 (ref. 71) scientific computing platform and the Spyder^72^ integrate development environment, where several numeric Python packages have been used, including NumPy, Pandas, SciPy and Matplotlib.^73–76^ The above computational program has been packaged and distributed as an open source Python code (SALAM).^77^

All quantum chemical computations is done by the Gaussian 16 package.^78^

## 3 Results and discussion

This work is mainly designed to examine the effect of different structural mutations as well as selection strategy on the result of HTVS of TADF materials. The initial compound library G_0_ is generated by combinatorial enumeration of 30 donors and 43 acceptors under the donor–acceptor (DA) molecular frameworks (details in ESI†). The kernel property adopted for optimization is the vertical energy gap Δ*E*_ST_.

### 3.1 Material abundance

**Fig. 3:**
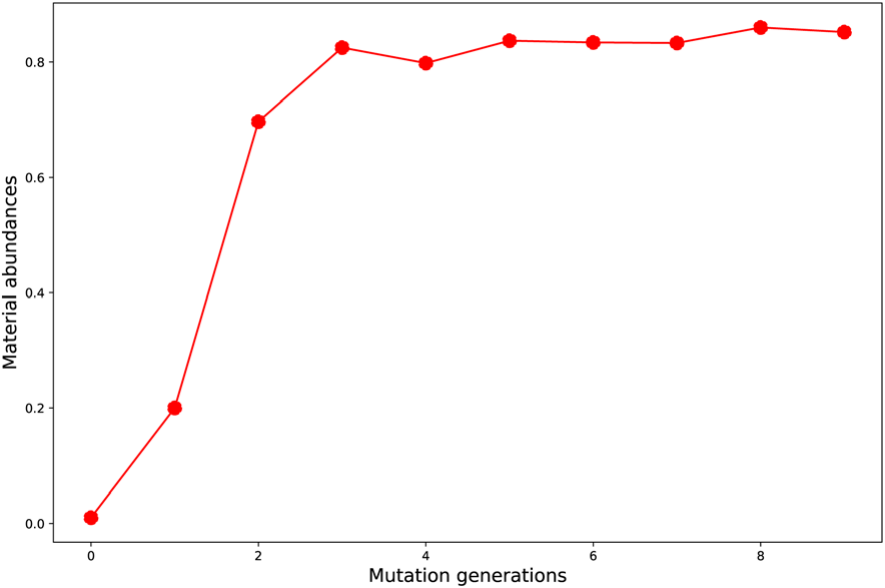
The material abundance *versus* mutation generation for **Sub3**.

Starting from the common baseline library (G_0_), the evolution of material abundance (*ω*_MA_) with the increase of mutation generation (*n*_g_) for different mutations has been listed in Table 1. For any of the studied mutations, the initial *ω*_MA_ is 0.010 (*n*_g_ = 0), as *n*_g_ increases, the *ω*_MA_ typically can display a sharp increase at low *n*_g_ values, and give slow increase at meadian *n*_g_ values, and finally trend to approach (or oscillate around) their respective limits. For an illustration of this behavior, the material abundance *versus* mutation generation for mutation = F (**Sub3**) is depicted in Fig. 3. From the rather low 0.010 (*n*_g_ = 0) increases to relatively high 0.825 (*n*_g_ = 3) and finally ceases at 0.852 (*n*_g_ = 9). For succeeded generations, the corresponding *ω*_MA_ may experience significant decrease, however, the overall trend to stay around a limit is rather obvious.

**Table 1:** The evolution of material abundance (*ω*_MA_) with increase of mutation generation (*n*_g_) for different mutations

*n*_g_	**Sub1**	**Sub2**	**Sub3**	**Sub4**	**Sub5**	**Sub6**	**Sub7**	**Sub8**	**Sub9**	**Sub10**
0	0.010	0.010	0.010	0.010	0.010	0.010	0.010	0.010	0.010	0.010
1	0.133	0.076	0.200	0.025	0.121	0.093	0.186	0.133	0.067	0.152
2	0.460	0.033	0.697	0.351	0.337	0.027	0.967	0.648	0.901	0.799
3	0.766	0.210	0.825	0.634	0.953	0.179	0.968	0.967	0.865	0.708
4	0.592	0.481	0.798	0.762	0.805	0.352	0.975	0.649	0.720	0.567
5	0.789	0.330	0.837	0.857	0.926	0.591	0.879	0.423	0.916	0.845
6	0.760	0.376	0.834	0.782	0.959	0.785	0.929	0.941	0.982	0.894
7	0.747	0.368	0.833	0.814	0.949	0.927	0.958	0.979	0.812	1.000
8	0.760	0.197	0.860	0.867	0.877	0.876	1.000	0.823	0.841	0.777
9	0.642	0.306	0.852	0.946	0.895	0.716	0.995	0.915	0.982	1.000

A comparison of **Sub1** and **Sub2** (correspond to different mutation speeds: slow *versus* fast), tells that for this type of mutations (aC–H → aN) a slow mutation speed is favorable than a fast one in achieving high *ω*_MA_ at large *n*_g_. As *n*_g_ = 9, the *ω*_MA_ for **Sub1** and **Sub2** are 0.642 and 0.306, correspondingly. For the second type of mutations (aC–H → aC–G), the evolution of *ω*_MA_ seem relatively small as *n*_g_ increases to high values. To sum up, except the fast mutation **Sub2**, all of the rest mutations can effectively concentrate ‘good’ molecules in compound library, hence give large *ω*_MA_ values.

### 3.2 Average number of aromatic aCH bonds (*n*_aCH_)

Turn back to the setup of the computational road map, it's natural to expect that as the two types mutations occupy positions (say, the mutation generation increases) the average number of aromatic aCH bonds of a compound library would experience significant drop in magnitude across the process. The evolution of average number of aromatic aCH bonds (*n*_aCH_) with increase of mutation generation (*n*_g_) for different mutations is listed in Table 2. It's noted that the overall evolution trend for *n*_aCH_ (uniformly decreases in value as *n*_g_ increases) is as we expected, though exceptions (alternative increase and decrease) do exist. Starting from the baseline 18.3 (*n*_g_ = 0), the *n*_aCH_ can drop down to a smaller value (generally lower than 10.0 as *n*_g_ = 9).

**Table 2:** The evolution of average number of aromatic aCH bonds (*n*_aCH_) with increase of mutation generation (*n*_g_) for different mutations

*n*_g_	**Sub1**	**Sub2**	**Sub3**	**Sub4**	**Sub5**	**Sub6**	**Sub7**	**Sub8**	**Sub9**	**Sub10**
0	18.3	18.3	18.3	18.3	18.3	18.3	18.3	18.3	18.3	18.3
1	15.7	14.8	14.9	14.9	14.9	14.9	15.8	15.9	15.8	15.7
2	13.7	11.1	11.3	10.0	12.9	10.7	13.1	12.6	14.7	12.8
3	11.8	7.8	10.1	11.1	9.4	8.9	17.8	10.5	13.8	14.5
4	11.9	6.5	6.3	12.5	8.0	15.6	11.1	9.3	16.6	15.1
5	11.1	5.5	6.2	10.9	6.9	19.2	10.7	13.4	19.2	20.1
6	11.1	5.7	5.4	7.9	7.2	18.1	11.3	6.1	14.0	21.1
7	11.2	5.8	5.3	8.0	5.9	20.2	10.5	4.8	10.5	20.4
8	10.9	5.5	5.0	7.8	6.3	18.2	10.6	4.4	7.8	4.0
9	10.8	5.4	4.9	8.2	5.4	16.0	9.7	3.6	7.5	2.9

**Fig. 4:**
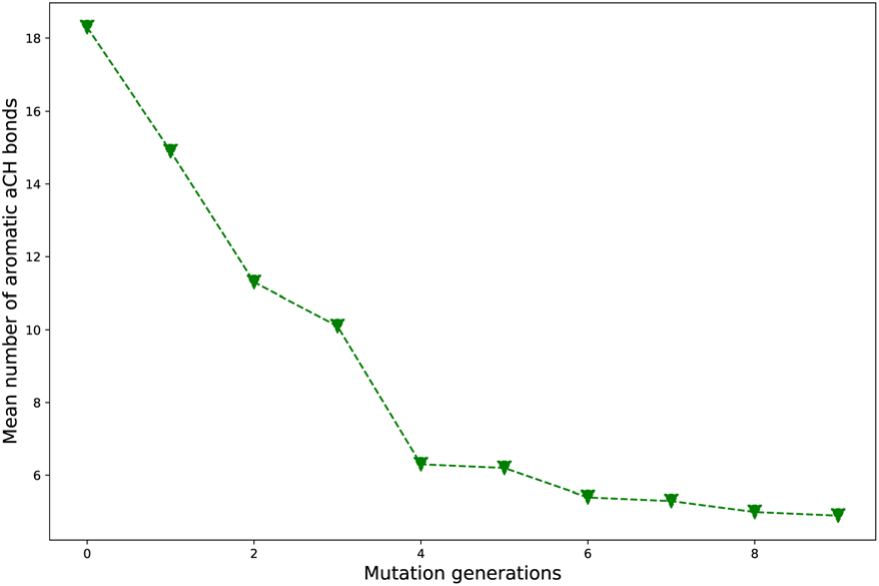
The mean number of aromatic aCH bonds *versus* mutation generation for **Sub3**.

Take **Sub3** as a case, whose *n*_aCH_ starts from a large value 18.3 (*n*_g_ = 0), sharply decreases to 6.3 (*n*_g_ = 4), finally drops to 4.9 (*n*_g_ = 9). The related diagram for **Sub3** has been depicted in Fig. 4. The starting large *n*_aCH_ should be attributed to large amount of unsubstituted molecules with multi-cyclic aromatic structures in the library. A small ending *n*_aCH_ should be attributed to large amount of oversubstituted molecules in the library, while a large ending *n*_aCH_ should be attributed to concentration of very large multi-cyclic aromatic molecules with low substitutions in the library. Thus, the seemingly anomalous phenomena of alternative increase and decrease in *n*_aCH_ can be understood by tracing the evolution of molecular skeletons of the compound library.

As compared with the slow mutation (**Sub1**), the *n*_aCH_ of the fast mutation (**Sub2**) exhibits a very rapid drop in value. However, this rapid drop in *n*_aCH_ is not sufficient to guarantee a meaningful increase in *ω*_MA_ (compare Tables 2 and 1). For some mutations (**Sub6** and **Sub10**), the anomalous alternative increase and decrease in *n*_aCH_ is a sign of violent transformation of dominant molecular skeletons under selection and mutation process. Therefore, it can be used as an indicator to differentiate the skeleton transformation effect of different mutations on the same DA substrates. If *n*_aCH_ retains large values as *n*_g_ turns large, there would be great amount of relatively ‘big’ molecules accumulated in library. Otherwise, if *n*_aCH_ exhibits rapid drop as *n*_g_ increases, there would be great amount of relatively ‘small’ molecules accumulated in library. Generally, from a point of view of synthetic chemistry, the ‘small’ molecules is more favorable than the ‘big’ ones.

To sum up, analysis of the *n*_aCH_ of different mutations tells us that the uniform drop in *n*_aCH_ with increase of *n*_g_ is not a sufficient condition to guarantee a meaningful increase in *ω*_MA_, rather it can be used a an indicator to differentiate different mutations on skeleton transformation effect.

### 3.3 Accumulated optimal molecules

Harvesting optimal molecules as more as possible by applying the designed HTVS program is one of the most important aims to be achieved. In other words, the best mutation should be the one which can induce the molecule's property in the right direction to fulfill the requirement as material. Since in the design of the selection and mutation computational step, the parent molecules are intrinsically added as part to form the new compound library, we think a number of accumulated optimal molecules along the mutation generations should be more suitable to account for the concentrating ability of the different mutations.

**Fig. 5:**
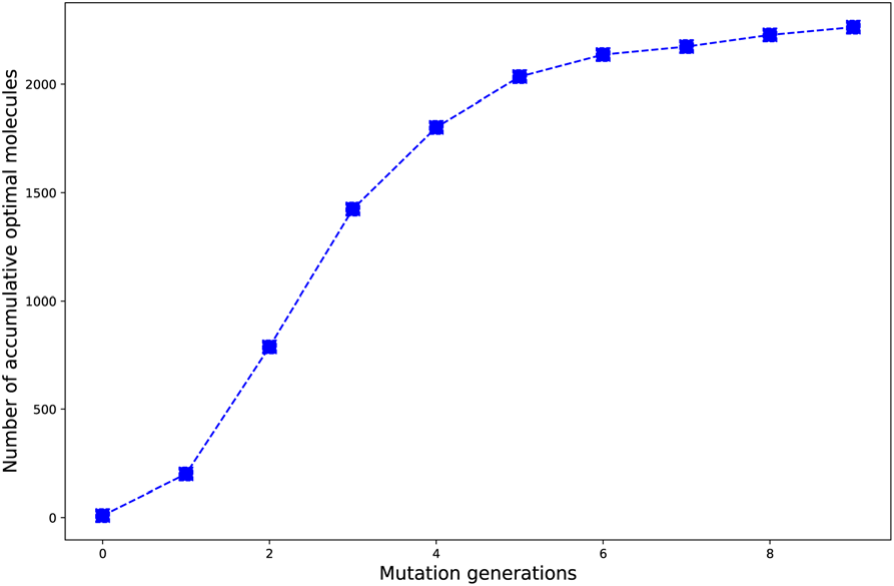
The number of accumulated optimal molecules *versus* mutation generation for **Sub3**.

The evolution of number of accumulated optimal molecules (*n*_acc_opt_mols_) with increase of mutation generation (*n*_g_) for different mutations has been listed in Table 3. The *n*_acc_opt_mols_ for any of mutations can exhibit a sharp increase in low to middle *n*_g_ values (0 < *n*_g_ ≤ 5), follow by slower growth for middle to high *n*_g_ (6 ≤ *n*_g_ ≤ 9), and may finally trend to flatten out. The probability of finding identical molecules between adjacent libraries will increase as *n*_g_ becomes large. This behavior is best demonstrated by the data of **Sub3**, as depicted in Fig. 5.

**Table 3:** The evolution of number of accumulated optimal molecules (*n*_acc_opt_mols_) with increase of mutation generation (*n*_g_) for different mutations

*n*_g_	**Sub1**	**Sub2**	**Sub3**	**Sub4**	**Sub5**	**Sub6**	**Sub7**	**Sub8**	**Sub9**	**Sub10**
0	10	10	10	10	10	10	10	10	10	10
1	83	83	202	34	125	97	191	137	75	156
2	288	108	791	341	418	122	1048	695	910	870
3	556	275	1429	807	1195	285	1894	1500	1679	1463
4	781	533	1808	1464	1607	576	2728	2043	2329	1947
5	1052	615	2041	2168	1841	1078	3455	2399	3143	2666
6	1345	696	2144	2667	2191	1768	4265	3104	4012	3453
7	1626	732	2182	3074	2380	2655	5083	3807	4730	4318
8	1896	741	2236	3433	2596	3423	5961	4328	5376	4645
9	2063	753	2273	3970	2730	4057	6833	4803	6172	4902

As compared with the fast mutation **Sub2**, the slow mutation **Sub1** can give approximately 2.7 times increase in *n*_acc_opt_mols_. Thus, **Sub1** is more favorable than **Sub2** in producing optimal molecules. Taking *n*_g_ = 9 as base, for the 4 terminal single mutations (**Sub3** to **Sub6**), the precedence order is: **Sub3** < **Sub5** < **Sub4** ≈ **Sub6**, a strong donor (or acceptor) is superior to a weak one; for the rest 4 mixed mutations (**Sub7** to **Sub10**), the precedence order is: **Sub8** < **Sub10** < **Sub9** < **Sub7**, the weak–weak pair exhibits superiority among others. The mixed mutations would produce more optimal molecules as expected since they correspond to larger chemical compound spaces, however, the price is the significant increase in molecular complexity, which might eventually prohibit them as material due to difficulty from experimental synthesis.

### Mean energy gap 
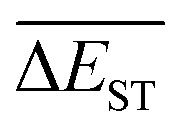


3.4

A successful HTVS program ought to effectively drive the optimized property to the optimal direction. For the current case, the evolution of mean energy gap 
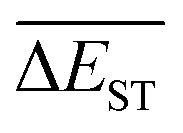
 of library along with the mutation generations has been listed in Table 4. It's observed that: the 
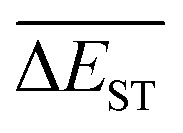
 for any of all mutations can exhibit a sharp decrease as *n*_g_ moves from low to middle values (0 < *n*_g_ ≤ 3), and trend to flatten out from middle to high values (3 < *n*_g_ ≤ 9). A large proportion of molecules with low Δ*E*_ST_ is enough to maintain the mean quantity 
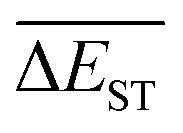
 of library at low value.

**Table 4:** The evolution of mean energy gap 
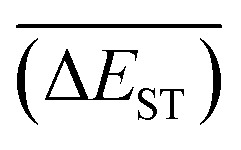
 with increase of mutation generation (*n*_g_) for different mutations

*n*_g_	**Sub1**	**Sub2**	**Sub3**	**Sub4**	**Sub5**	**Sub6**	**Sub7**	**Sub8**	**Sub9**	**Sub10**
0	0.669	0.669	0.669	0.669	0.669	0.669	0.669	0.669	0.669	0.669
1	0.292	0.369	0.246	0.332	0.280	0.310	0.262	0.298	0.253	0.264
2	0.218	0.316	0.140	0.218	0.275	0.236	0.065	0.150	0.096	0.115
3	0.088	0.276	0.098	0.139	0.086	0.331	0.046	0.134	0.103	0.116
4	0.187	0.207	0.095	0.116	0.106	0.178	0.051	0.144	0.121	0.151
5	0.104	0.268	0.078	0.101	0.086	0.165	0.082	0.256	0.098	0.085
6	0.120	0.233	0.075	0.108	0.085	0.124	0.058	0.052	0.057	0.078
7	0.110	0.249	0.076	0.102	0.084	0.076	0.050	0.066	0.108	0.100
8	0.114	0.274	0.074	0.077	0.089	0.104	0.042	0.092	0.090	0.096
9	0.155	0.229	0.076	0.059	0.085	0.128	0.043	0.073	0.063	0.043

**Fig. 6:**
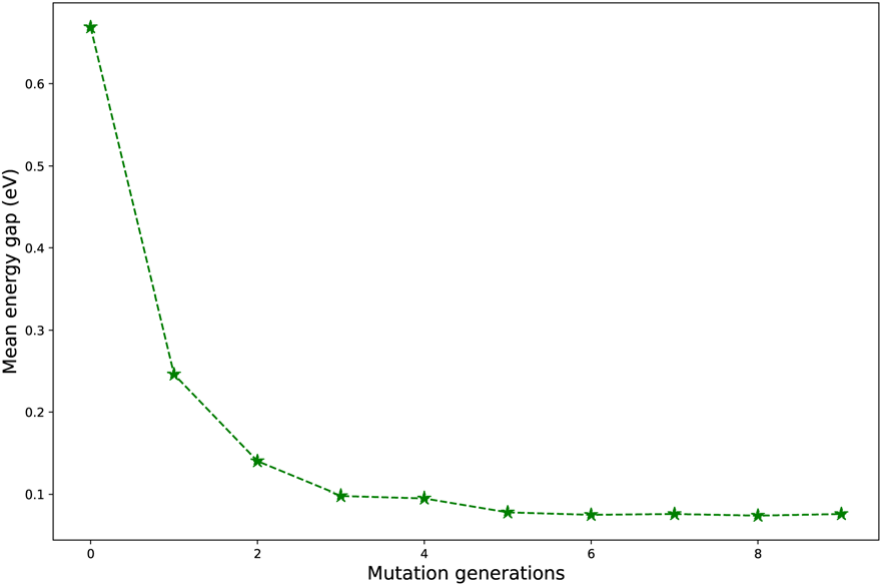
The evolution of mean energy gaps *versus* mutation generation for **Sub3**.

Regardless of the types of mutations, the fast convergent trend of 
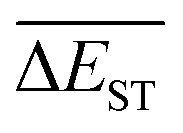
 is rather impressive. The evolution of mean energy gaps *versus* mutation generation for **Sub3** has been depicted in Fig. 6. The 
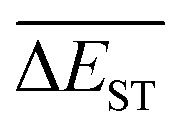
 starts from a value of 0.669 eV (*n*_g_ = 0), experiences a sharp drop to 0.098 eV (*n*_g_ = 3), finally trends to flatten out to 0.076 eV (*n*_g_ = 9). There should have a clear correlation between the evolutionary behaviors of 
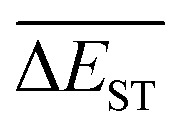
 and *ω*_MA_, since both of them are group quantities based on the Δ*E*_ST_ of molecules.

**Fig. 7:**
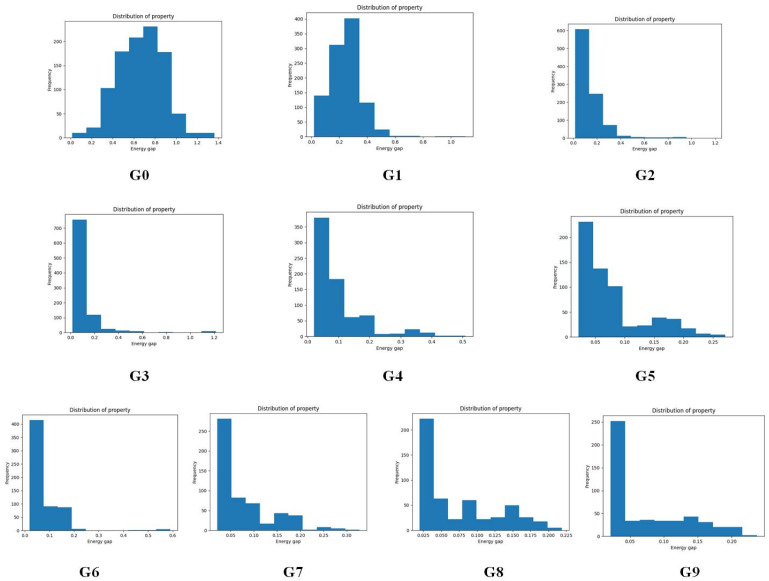
The evolution of energy gaps frequency distribution *versus* mutation generation for **Sub3**.

To give more details on the impact of the mutation along with mutation generations, the evolution of energy gaps frequency distribution *versus* mutation generation for **Sub3** has been depicted in Fig. 7. The fast shift to low Δ*E*_ST_ is apparent (*n*_g_ moves from 0 to 3), then the distribution retains a large proportion in the very low value range and tails in the low to medium range.

To sum up, regardless of the types of mutations, the mean energy gap can exhibit a fast convergent trend toward very low values, hence the studied mutations (except **Sub2**) can cooperate very well with the DA substrates to generate optimal molecules.

### 3.5 Skeleton decomposition

Decomposition of a closely connected compound library into molecular skeletons is an effective way to examine it's main skeleton compositions. Considering the loop nature of the designed HTVS program, it's interesting to observe how the dominant molecular skeletons can evolve with the increase of mutation generation. By applying the Murcko Skeleton Decomposition method, the related data has been computed. Two types of molecular skeletons (common and generic cores) are adopted. The common cores identify both types of elements and bonds, while the generic cores neglect this information.

**Fig. 8:**
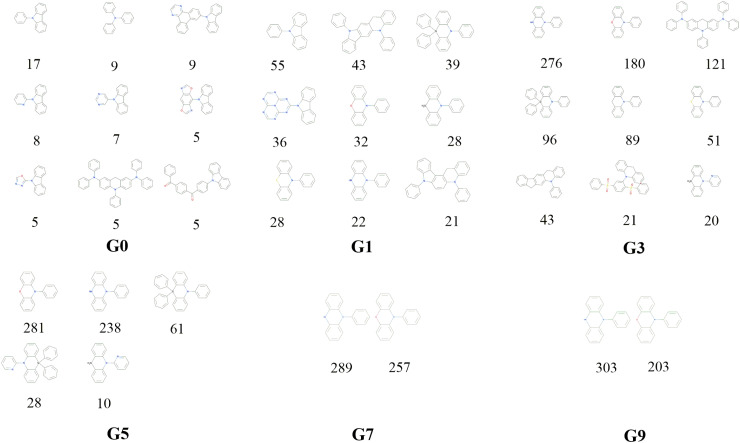
The evolution of skeleton (core) with mutation generation for **Sub3** (the numbers below structures denote the corresponding frequencies).

The evolution of skeletons (common cores) *versus* mutation generation for **Sub3** has been depicted in Fig. 8, and that of skeletons (generic cores) has been given in Fig. S4 in the ESI.† For simplicity, at most 9 dominant high-frequency skeletons and selected mutation generations have been shown. For both types of cores, there exists explicit quantitative shrinkage of dominant high-frequency skeletons. The common cores starts from the relatively uniform distribution of frequencies of 9 cores (*n*_g_ = 0), then collapses to very uneven distribution of frequencies of 5 cores (*n*_g_ = 5), and finally collapses further to a distribution of frequencies of only 2 cores (*n*_g_ = 9) (Fig. 8). The generic cores can exhibit more profound collapse in number of cores (Fig. S4 in the ESI†). This collapse is a sign of efficient convergence of the structures around ‘excellent’ molecules. Hence is beneficial for obtaining optimal molecules.

### 3.6 Similarity analysis between mutation generations

Similarity is an important concept used to describe the degree of difference in the structure of two molecules. Using this concept, and introducing a valid fingerprint representation for molecule and a similarity metric, the similarity can be easily calculated for any pair of two molecules.

Following the concept of similarity for two molecules and based on molecular fingerprint representation, we propose a numerical method to calculate group fingerprint similarity (Δ_MSPR_) between two compound libraries. Here, the molecular fingerprint representation method is ECFP, and the similarity is measured by the Tanimoto metric.

The calculation of the group fingerprint similarity (Δ_MSPR_) is based a algorithm, which we name it the Maximum Similarity Pairing Rule (MSPR) (refer to ESI†). The evolution of the number of molecules in library (*n*_tot_), the number of intersection molecules (*n*_inter_), and the group fingerprint similarity between two libraries (Δ_MSPR_) with increase of mutation generation (*n*_g_) for **Sub1**, **Sub3** and **Sub7** is listed in Table 5.

**Table 5:** The evolution of the number of molecules in library (*n*_tot_), the number of intersection molecules (*n*_inter_), and the group fingerprint similarity between two libraries (Δ_MSPR_) with increase of mutation generation (*n*_g_) for **Sub1**, **Sub3** and **Sub7**

*n*_g_	**Sub1**			**Sub3**			**Sub7**		
	*n*_tot_	*n*_inter_	Δ_MSPR_	*n*_tot_	*n*_inter_	Δ_MSPR_	*n*_tot_	*n*_inter_	Δ_MSPR_
0	1000	—	—	1000	—	—	1000	—	—
1	588	100^a^	0.660^a^	1000	100	0.542	1000	98	0.553
2	531	125	0.738	960	111	0.638	1000	127	0.666
3	487	127	0.778	929	140	0.787	1000	122	0.737
4	585	179	0.900	747	227	0.893	1000	135	0.735
5	541	161	0.834	618	282	0.949	1000	171	0.834
6	570	151	0.872	608	354	0.908	1000	126	0.779
7	588	180	0.898	546	350	0.932	1000	138	0.910
8	574	173	0.930	514	339	0.980	1000	120	0.891
9	565	212	0.928	506	364	0.992	1000	117	0.959

a Calculated with respect to the corresponding precedent *n*_g_.

For **Sub1**, the *n*_tot_ keeps a size of about 500 for *n*_g_ in range from 1 to 9, and the *n*_inter_ exhibits a slowly increase trend in that range, hence the Δ_MSPR_ can change from a low value of 0.660 (for *n*_g_ = 1) to a high value 0.928 (for *n*_g_ = 9). For **Sub3**, the rather high values of Δ_MSPR_ for high mutation generations (*n*_g_ ≥ 5) can be ascribed by the ordered descending of *n*_tot_ and incrementing of *n*_inter_. For **Sub7**, both of *n*_tot_ and *n*_inter_ keep their sizes as *n*_g_ increases. The high values of Δ_MSPR_ for high mutation generations (*n*_g_ ≥ 7) can be ascribed by the convergence in molecular skeletons, which can retain the similarity between pairs of molecules at high values of range.

To sum up, regardless of types of mutations, the group fingerprint similarity (Δ_MSPR_) at high mutation generations can retain high enough values (typically larger than 0.90), which can be associated with the apparent convergence in molecular skeletons at high mutation generations.

### 3.7 Optimal TADF molecules (low Δ*E*_ST_ and SAS)

**Fig. 9:**
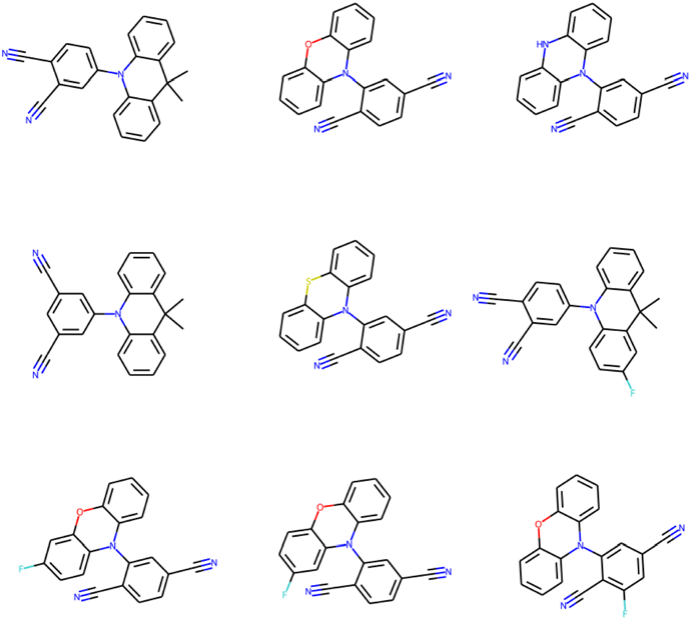
The structures of 9 optimal molecules with lowest SAS for **Sub3**.

The (energy gap) optimal molecules for all mutations have been sorted by synthetic accessibility scores from low to high, so as to give recommendation for TADF materials candidates. The structures of 9 molecules with lowest SAS for **Sub3** have been depicted in Fig. 9. For other mutations, the related structures are given by Fig. S5 in the ESI.†

In principle, molecules with simpler and more symmetric structures are favored by the SAS sorting routine. Within the studied compound space, those compounds constructed by typical tri-cyclic donors connecting with (polyacetonitrile substituted) benzenes acceptors can possess the lowest SAS. In addition, they can exhibit low enough energy gaps. Therefore, from the point of view of synthetic chemistry, they are recommended as optimal TADF molecules (low Δ*E*_ST_ and SAS), although only a small energy gap might not be enough to guarantee the occurrence of TADF emission.

Notably, possessing a low enough Δ*E*_ST_ as a necessary condition, the real occurrence of TADF emission for a compound should at least be accompanied with an acceptable radioactive fluorescent rate. Since the number of accumulated optimal molecules for high mutation generation are typically larger than 2000, we expect the extra fulfillment of the radioactive fluorescent rate may have great chance to occur possibly by further sieving the already obtained compound library of accumulated optimal molecules.

### 3.8 Recommendation for combinations of DA substrates with mutations

#### 3.8.1 Optimal molecular skeletons for realizing low energy gaps

Regardless of the mutations, the high material abundances (typically >0.8) at large mutation generations (*n*_g_ ≥ 6) naturally indicate the existence of ‘optimal’ molecular skeletons for realizing low energy gaps as substituted by suitable mutations. The SMILES of common and generic cores, and their frequencies for studied mutations as *n*_g_ = 9 have been listed in Table 6.

**Table 6:** The SMILES of common and generic cores, and their frequencies for studied mutations as *n*_g_ = 9

Mutations		Freq
**SMILES of common cores**		
**Sub3**	c1ccc(N2c3ccccc3Nc3ccccc32)cc1	303
	c1ccc(N2c3ccccc3Oc3ccccc32)cc1	203
**Sub4**	O=S(=O)(c1ccccc1)c1ccc(S(=O)(=O)c2ccccc2)c(N2c3ccccc3Cc3ccccc32)c1	640
	O=S1(=O)c2ccccc2S(=O)(=O)c2cc(N3c4ccccc4Cc4ccccc43)ccc21	122
	O=S1(=O)c2ccccc2S(=O)(=O)c2cc(N3c4ccccc4Oc4ccccc43)ccc21	80
	c1ccc(N2c3ccccc3Oc3ccccc32)cc1	29
**Sub5**	c1ccc(N2c3ccccc3Nc3ccccc32)cc1	243
	c1ccc(N2c3ccccc3Cc3c2ccc2c3c3ccccc3n2-c2ccccc2)cc1	233
**Sub6**	c1ccc(N(c2ccccc2)c2ccc3c(c2)Cc2cc(N(c4ccccc4)c4ccccc4)ccc2N3c2ccccc2)cc1	1000
**Sub7**	c1ccc(N2c3ccccc3Cc3cc4c(cc32)c2ccccc2n4-c2ccccc2)cc1	984
	c1ccc(N2c3ccccc3Sc3ccccc32)cc1	16
**Sub8**	c1ccc(N2c3ccccc3Nc3ccccc32)cc1	662
	c1ccc(N2c3ccccc3Oc3ccccc32)cc1	134
**Sub9**	c1ccc(N2c3ccccc3Cc3cc4c(cc32)c2ccccc2n4-c2ccccc2)cc1	688
	c1ccc(N2c3ccccc3Cc3ccccc32)cc1	176
	c1ccc(N2c3ccccc3Cc3cc4c(cc32)Cc2ccccc2-4)cc1	136
**Sub10**	c1ccc(N2c3ccccc3Nc3ccccc32)cc1	555

**SMILES of generic cores**		
**Sub1**	C1CCC(C2C3CCCCC3CC3CC4C(CC32)C2CCCCC2C4C2CCCCC2)CC1	565
**Sub2**	C1CCC(C2C3CCCCC3CC3CCCCC32)CC1	506
	C1CCC(C2C3CCCCC3CC3CC4C(CC5CCCCC54)CC32)CC1	170
**Sub3**	C1CCC(C2C3CCCCC3CC3CCCCC32)CC1	506
**Sub4**	CC(C)(C1CCCCC1)C1CCC(C(C)(C)C2CCCCC2)C(C2C3CCCCC3CC3CCCCC32)C1	640
	CC1(C)C2CCCCC2C(C)(C)C2CC(C3C4CCCCC4CC4CCCCC43)CCC21	202
	C1CCC(C2C3CCCCC3CC3CCCCC32)CC1	29
**Sub5**	C1CCC(C2C3CCCCC3CC3CCCCC32)CC1	243
	C1CCC(C2C3CCCCC3CC3C2CCC2C(C4CCCCC4)C4CCCCC4C32)CC1	233
**Sub6**	C1CCC(C(C2CCCCC2)C2CCC3C(CC4CC(C(C5CCCCC5)C5CCCCC5)CCC4C3C3CCCCC3)C2)CC1	1000
**Sub7**	C1CCC(C2C3CCCCC3CC3CC4C(CC32)C2CCCCC2C4C2CCCCC2)CC1	984
	C1CCC(C2C3CCCCC3CC3CCCCC32)CC1	16
**Sub8**	C1CCC(C2C3CCCCC3CC3CCCCC32)CC1	796
**Sub9**	C1CCC(C2C3CCCCC3CC3CC4C(CC32)C2CCCCC2C4C2CCCCC2)CC1	688
	C1CCC(C2C3CCCCC3CC3CCCCC32)CC1	176
	C1CCC(C2C3CCCCC3CC3CC4C(CC5CCCCC54)CC32)CC1	136
**Sub10**	C1CCC(C2C3CCCCC3CC3CCCCC32)CC1	555

The distribution of frequencies of different skeletons for a specific mutation is generally uneven with one dominant skeleton. The common core with SMILES = “c1ccc(N2c3ccccc3Nc3ccccc32)cc1” exists for several mutations (**Sub3**, **Sub5**, **Sub8** and **Sub10**) with associated frequencies (303, 243, 662 and 555). The common core with SMILES = “c1ccc(N2c3ccccc3Oc3ccccc32)cc1” exists for several mutations (**Sub3**, **Sub4** and **Sub8**) with associated frequencies (203, 29 and 134). The common core with SMILES = “c1ccc(N2c3ccccc3Cc3cc4c(cc32)c2ccccc2n4-c2ccccc2)cc1” exists for two mutations (**Sub7** and **Sub9**) with associated frequencies (984 and 688). Similarly, the common core with SMILES = “O=S(=O)(c1ccccc1)c1ccc(S(=O)(=O)c2ccccc2)c(N2c3ccccc3Cc3ccccc32)c1” exists only for **Sub4** with frequencies = 640. Different skeletons can exhibit distinguishable preference to associate with different mutations. In short, the mutation can select the optimal skeleton(s) out from thousands of original DA substrates to realize low energy gaps.

**Fig. 10:**
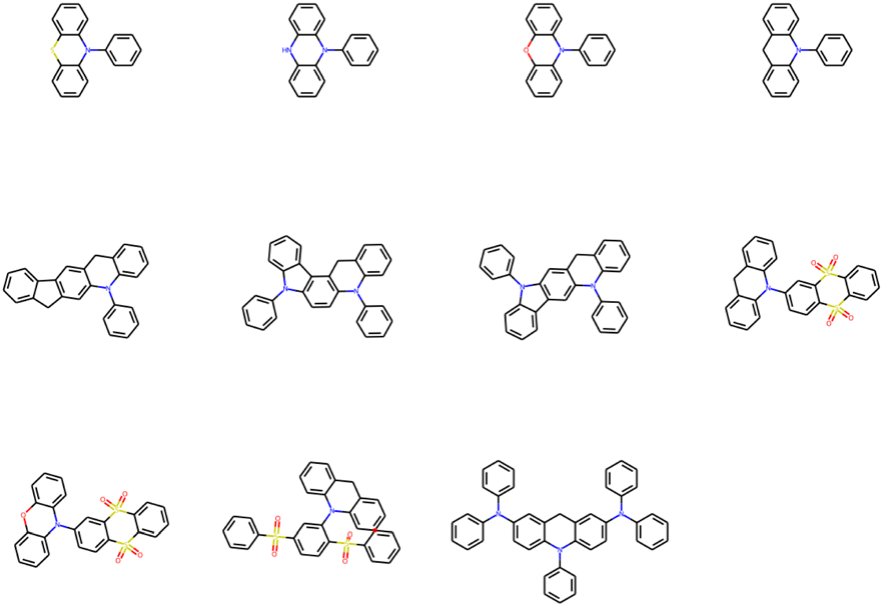
The structures of optimal skeletons (common cores) for mutations from **Sub3** to **Sub10** as *n*_g_ = 9.

After removing duplicates, the structures of optimal skeletons (common cores) for mutations from **Sub3** to **Sub10** as *n*_g_ = 9 have been depicted in Fig. 10. The related diagram for generic cores is given by Fig. S6 in ESI.† By definitions of common and generic cores, the common cores for **Sub1** and **Sub2** cannot collapse, however, their generic cores do belong to 1 or 2 skeletons (Table 6). It's interesting to note that the overall numbers of common and generic cores for all mutations are 11 and 7. Hence, in a sense, the ‘optimal’ skeletons seem unique and useful in realizing low energy gaps.

#### 3.8.2 Emitting colors

The optimization of structures is designed for one kernel property (the energy gap), hence the accompanied emitting color (energy) should not be optimized (as mutation generation increases). Conceptually, clean (with limited substitution) DA compounds should mainly exhibit blue emission owing to the intrinsic π → π* transition pattern. As hetero-atoms doping in the structure, there would be considerable red shift to occur. In addition, the connection of strong electronic donor to conjugate aromatic system would have effect to red-shift the emission. For the chosen DA substrates and mutations, analysis of data from the energy sieve step shows that:

(1) To access red color, **Sub10** (CN or NMe_2_) is the best mutation groups, which can produce considerable proportion red molecules when the mutation generation equals to 9.

(2) To access green color, **Sub1** (N(slow)), **Sub2** (N(fast)), **Sub4** (CN), **Sub9** (CN or OMe), and **Sub10** (CN or NMe_2_) are favored groups.

(3) To access blue color, all mutations seem valid. **Sub3** (F) and **Sub5** (OMe) are the recommended groups.

### 3.9 Perspective on the applicability of the designed HTVS program

By now, we have demonstrated the ability of the designed HTVS program to sieve organic TADF molecules (and skeletons) out by providing a set of preset mutations and starting from a medium size of compound library (10^3^ DA molecules) constructed by enumeration of donors and acceptors. By the design of the mutations (only conjugated aromatic units containing molecular systems are valid), once the mutations could occur within a new compound library, the electronic properties could effectively be modulated by the preset mutations, then the designed HTVS program would have great chance to be applicable to accumulate ‘good molecules' for specific kind of properties. Accordingly, upon certain modifications of the code (*e.g.,* adding more property analysis functions), we expect the designed HTVS program might have the following possible fields of applications:

* Systems: organic molecules within DA, D–π–A, D–A–D, A–D–A and D_3_–A frameworks; organometallic complexes with conjugate organic aromatic ligands.

* Properties: electronic and electric properties based on ground state (and possibly excited state) geometry of molecule.

* Materials: organic and organometallic TADF, nonlinear optical and two-photon absorption materials; and possibly organic conductive and photovoltaic materials.

Obviously, there are some areas for further improvement. Whether other types of mutations are possible for conjugated aromatic systems, and whether it is possible to design a valid crossover operator to combine two parent molecules to give offsprings molecules, have not been explored yet. Moreover, the program is driven by only one core property, sometimes there would be several properties to be optimized simultaneously, therefore, more design efforts should be devoted to support this kind of requirement. And, there should be more supporting on (artificial neural network based) deep learning methods to improve the models' accuracy for property prediction. Additionally, more quantum chemical packages as computing engines for electronic structure should be supported. Accompanied with the above-mentioned areas for improvement, we still hope that the designed HTVS programs could provide some valuable insights into related fields.

## 4 Conclusion

By combining machine learning and quantum chemical calculations, using cheminformatics tools, and introducing the concept of selection and mutation from evolutionary theory, we designed a computational program for high-throughput virtual screening of thermally activated delayed fluorescence molecular materials, especially the impact of selection strategy and structural mutations on the results of HTVS was explored. The energy gap was chosen as the kernel property to be optimized. The initial compounds library (DA substrates) was generated by combinatorial enumeration of fragments; 10 mutations was used; the Random Forest Regressor was adopt as the ML algorithm to be learned; a 10% ratio was set to randomly pick molecules from library to form the training set, and their geometries and electronic properties was computed by Gaussian; the molecular structure was featurized by the ECFP fingerprint method; by searching hyper-parameter space with 5-fold cross validation, along with the computed property, the training data was fed to the ML algorithm to obtain the best ML model; then the best ML model was used to predict unseen molecules in library. We have found that the mix of selection and mutations into the evolution map can have great impact on the HTVS results:

(1) Except the fast mutation **Sub2**, all of the rest mutations can effectively concentrate ‘good’ molecules in compound library, hence give large *ω*_MA_ (typically >0.8) for mutation generation at high values (*n*_g_ ≥ 6).

(2) Analysis of the *n*_aCH_ of different mutations tells us that the uniform drop in *n*_aCH_ with increase of *n*_g_ is not a sufficient condition to guarantee a meaningful increase in *ω*_MA_, rather it can be used a an indicator to differentiate different mutations on skeleton transformation effect.

(3) The *n*_acc_opt_mols_ for any of mutations can exhibit a sharp increase in low to middle *n*_g_ values (0 < *n*_g_ ≤ 5), follow by slower growth for middle to high *n*_g_ (6 ≤ *n*_g_ ≤ 9), and may finally trend to flatten out. **Sub1** is more favorable than **Sub2** in producing optimal molecules. For the 4 terminal single mutations, the precedence order is: **Sub3** < **Sub5** < **Sub4** ≈ **Sub6**; for the rest 4 mixed mutations, the precedence order is: **Sub8** < **Sub10** < **Sub9** < **Sub7**. The mixed mutations would produce more optimal molecules as expected in price of significant increase in molecular complexity, which might eventually prohibit them as material due to difficulty from experimental synthesis.

(4) The 
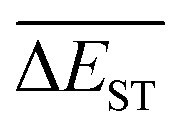
 can exhibit a fast convergent trend toward very low values, hence the studied mutations (except **Sub2**) can cooperate very well with the DA substrates to generate optimal molecules.

(5) A group fingerprint similarity (Δ_MSPR_) index was proposed to account for the similarity between two compound libraries with comparable sizes. The Δ_MSPR_ can retain high enough values (typically larger than 0.90) for large *n*_g_, which can be associated with the apparent convergence in molecular skeletons at high mutation generations.

(6) The distribution of frequencies of different skeletons for a specific mutation is generally uneven with one dominant skeleton. The overall numbers of common and generic cores for all mutations are 11 and 7 as *n*_g_ = 9. Hence, in a sense, the ‘optimal’ skeletons seem unique and useful in realizing low energy gaps.

With above observations and the development of HTVS software, we expect to provide insight and tool to the research community of HTVS of molecular (TADF) materials.

## Author contributions

C. Tu contributed to the conception of the study, designed the computational code, conducted the theoretical studies and data analysis, and drafted the manuscript; W. Huang contributed to the design of compound library; S. Liang contributed to the programming of the Python code; K. Wang and Q. Tian contributed to the conceptual and functional design of the computational code; W. Yan contributed to the conception of the study, revised the manuscript and supervised the whole work.

## Conflicts of interest

There are no conflicts to declare.

## Supplementary Material

RA-012-D2RA05643G-s001

## References

[cit1] Tang C. W., VanSlyke S. A. (1987). Appl. Phys. Lett..

[cit2] Adachi C. (2014). Jpn. J. Appl. Phys..

[cit3] Im Y., Kim M., Cho Y. J., Seo J.-A., Yook K. S., Lee J. Y. (2017). Chem. Mater..

[cit4] Highly Efficient OLEDs: Materials Based on Thermally Activated Delayed Fluorescence, ed. H. Yersin, Wiley-VCH Verlag GmbH & Co. KGaA, Weinheim, Germany, 2018

[cit5] Uoyama H., Goushi K., Shizu K., Nomura H., Adachi C. (2012). Nature.

[cit6] Zhang Q., Li B., Huang S., Nomura H., Tanaka H., Adachi C. (2014). Nat. Photonics.

[cit7] Hirata S., Sakai Y., Masui K., Tanaka H., Lee S. Y., Nomura H., Nakamura N., Yasumatsu M., Nakanotani H., Zhang Q., Shizu K., Miyazaki H., Adachi C. (2015). Nat. Mater..

[cit8] Zhang Q., Tsang D., Kuwabara H., Hatae Y., Li B., Takahashi T., Lee S. Y., Yasuda T., Adachi C. (2015). Adv. Mater..

[cit9] Cui L.-S., Nomura H., Geng Y., Kim J. U., Nakanotani H., Adachi C. (2017). Angew. Chem., Int. Ed..

[cit10] Penfold T. J. (2015). J. Phys. Chem. C.

[cit11] Etherington M. K., Gibson J., Higginbotham H. F., Penfold T. J., Monkman A. P. (2016). Nat. Commun..

[cit12] Etherington M. K., Franchello F., Gibson J., Northey T., Santos J., Ward J. S., Higginbotham H. F., Data P., Kurowska A., Dos Santos P. L., Graves D. R., Batsanov A. S., Dias F. B., Bryce M. R., Penfold T. J., Monkman A. P. (2017). Nat. Commun..

[cit13] Föller J., Kleinschmidt M., Marian C. M. (2016). Inorg. Chem..

[cit14] Gibson J., Monkman A. P., Penfold T. J. (2016). ChemPhysChem.

[cit15] Gómez-Bombarelli R., Aguilera-Iparraguirre J., Hirzel T. D., Duvenaud D., Maclaurin D., Blood-Forsythe M. A., Chae H. S., Einzinger M., Ha D.-G., Wu T., Markopoulos G., Jeon S., Kang H., Miyazaki H., Numata M., Kim S., Huang W., Hong S. I., Baldo M., Adams R. P., Aspuru-Guzik A. (2016). Nat. Mater..

[cit16] Peng Q., Fan D., Duan R., Yi Y., Niu Y., Wang D., Shuai Z. (2017). J. Phys. Chem. C.

[cit17] Samanta P. K., Kim D., Coropceanu V., Brédas J.-L. (2017). J. Am. Chem. Soc..

[cit18] Mewes J.-M. (2018). Phys. Chem. Chem. Phys..

[cit19] Olivier Y., Sancho-Garcia J.-C., Muccioli L., D'Avino G., Beljonne D. (2018). J. Phys. Chem. Lett..

[cit20] Penfold T. J., Dias F., Monkman A. P. (2018). Chem. Commun..

[cit21] Gao Y.-J., Chen W.-K., Wang Z.-R., Fang W.-H., Cui G. (2018). Phys. Chem. Chem. Phys..

[cit22] de Silva P., Kim C. A., Zhu T., Van Voorhis T. (2019). Chem. Mater..

[cit23] Kim I., Jeon S. O., Jeong D., Choi H., Son W.-J., Kim D., Rhee Y. M., Lee H. S. (2020). J. Chem. Theory Comput..

[cit24] Shafikov M. Z., Suleymanova A. F., Czerwieniec R., Yersin H. (2017). Chem. Mater..

[cit25] Yersin H., Mataranga-Popa L., Czerwieniec R., Dovbii Y. (2019). Chem. Mater..

[cit26] Parr R. G. and Yang W., Density-Functional Theory of Atoms and Molecules, Oxford University Press, 1989

[cit27] Runge E., Gross E. K. U. (1984). Phys. Rev. Lett..

[cit28] Morgan H. L. (1965). J. Chem. Doc..

[cit29] Weininger D. (1988). J. Chem. Inf. Model..

[cit30] Bemis G. W., Murcko M. A. (1996). J. Med. Chem..

[cit31] Lewell X. Q., Judd D. B., Watson S. P., Hann M. M. (1998). J. Chem. Inf. Comput. Sci..

[cit32] Degen J., Wegscheid-Gerlach C., Zaliani A., Rarey M. (2008). ChemMedChem.

[cit33] Rogers D., Hahn M. (2010). J. Chem. Inf. Model..

[cit34] Landrum G. , The RDKit Documentation—The RDKit 2020.09.1 documentation, 2020, http://www.rdkit.org/docs/index.html

[cit35] Riniker S., Landrum G. A. (2015). J. Chem. Inf. Model..

[cit36] Moriwaki H., Tian Y.-S., Kawashita N., Takagi T. (2018). J. Cheminf..

[cit37] Turcani L., Berardo E., Jelfs K. E. (2018). J. Comput. Chem..

[cit38] Pyzer-Knapp E. O., Suh C., Gómez-Bombarelli R., Aguilera-Iparraguirre J., Aspuru-Guzik A. (2015). Annu. Rev. Mater. Res..

[cit39] Wilbraham L., Berardo E., Turcani L., Jelfs K. E., Zwijnenburg M. A. (2018). J. Chem. Inf. Model..

[cit40] Ma X.-Y., Lewis J. P., Yan Q.-B., Su G. (2019). J. Phys. Chem. Lett..

[cit41] Pedregosa F., Varoquaux G., Gramfort A., Michel V., Thirion B., Grisel O., Blondel M., Prettenhofer P., Weiss R., Dubourg V., Vanderplas J., Passos A., Cournapeau D. (2011). J. Mach. Learn. Res..

[cit42] Amr T. , Hands-On Machine Learning with Scikit-learn and Scientific Python Toolkits, Packt Publishing Ltd, Birmingham, 2020

[cit43] Abadi M. , Barham P., Chen J., Chen Z., Davis A., Dean J., Devin M., Ghemawat S., Irving G., Isard M., Kudlur M., Levenberg J., Monga R., Moore S., Murray D. G., Steiner B., Tucker P., Vasudevan V., Warden P., Wicke M., Yu Y. and Zheng X., 12th USENIX Symposium on Operating Systems Design and Implementation (OSDI 16), Savannah, GA, 2016, pp. 265–283

[cit44] Paszke A., Gross S., Massa F., Lerer A., Bradbury J., Chanan G., Killeen T., Lin Z., Gimelshein N., Antiga L., Desmaison A., Kopf A., Yang E., DeVito Z., Raison M., Tejani A., Chilamkurthy S., Steiner B., Fang L., Bai J., Chintala S. (2019). Adv. Neural Inf. Process. Syst..

[cit45] Wu Z., Ramsundar B., Feinberg E. N., Gomes J., Geniesse C., Pappu A. S., Leswing K., Pande V. (2018). Chem. Sci..

[cit46] Ramsundar B. , Eastman P., Walters P., Pande V., Leswing K. and Wu Z., Deep Learning for the Life Sciences, O'Reilly Media, 2019

[cit47] Dral P. O. (2019). J. Comput. Chem..

[cit48] Jacobs R., Mayeshiba T., Afflerbach B., Miles L., Williams M., Turner M., Finkel R., Morgan D. (2020). Comput. Mater. Sci..

[cit49] Butler K. T., Davies D. W., Cartwright H., Isayev O., Walsh A. (2018). Nature.

[cit50] Rupp M., Tkatchenko A., Müller K.-R., von Lilienfeld O. A. (2012). Phys. Rev. Lett..

[cit51] Snyder J. C., Rupp M., Hansen K., Müller K.-R., Burke K. (2012). Phys. Rev. Lett..

[cit52] Ramakrishnan R. and von Lilienfeld O. A., Reviews in Computational Chemistry, John Wiley & Sons, Inc., 2017, pp. 225–256

[cit53] Ramakrishnan R., Dral P. O., Rupp M., von Lilienfeld O. A. (2015). J. Chem. Theory Comput..

[cit54] Lilienfeld O. A. v., Müller K.-R., Tkatchenko A. (2020). Nat. Rev. Chem..

[cit55] Huang B., von Lilienfeld O. A. (2020). Nat. Chem..

[cit56] Ramakrishnan R., Dral P. O., Rupp M., von Lilienfeld O. A. (2014). Sci. Data.

[cit57] Ye S., Hu W., Li X., Zhang J., Zhong K., Zhang G., Luo Y., Mukamel S., Jiang J. (2019). Proc. Natl. Acad. Sci..

[cit58] Ma S., Liu Z.-P. (2020). ACS Catal..

[cit59] Chen W.-K., Liu X.-Y., Fang W.-H., Dral P. O., Cui G. (2018). J. Phys. Chem. Lett..

[cit60] Kingma D. P. and Welling M., Auto-Encoding Variational Bayes, *arXiv*, 2014, preprint, arXiv:1312.6114

[cit61] Gómez-Bombarelli R., Wei J. N., Duvenaud D., Hernández-Lobato J. M., Sánchez-Lengeling B., Sheberla D., Aguilera-Iparraguirre J., Hirzel T. D., Adams R. P., Aspuru-Guzik A. (2018). ACS Cent. Sci..

[cit62] Holland J. H. , Adaptation in natural and artificial systems: an introductory analysis with applications to biology, control, and artificial intelligence, MIT press, 1992

[cit63] Stewart J. J. (2007). J. Mol. Model..

[cit64] Grimme S., Antony J., Ehrlich S., Krieg H. (2010). J. Chem. Phys..

[cit65] Chai J.-D., Head-Gordon M. (2008). Phys. Chem. Chem. Phys..

[cit66] Calculate Root-mean-square deviation (RMSD) of Two Molecules Using Rotation, version 1.4, https://github.com/charnley/rmsd

[cit67] Kabsch W. (1976). Acta Crystallogr., Sect. A: Cryst. Phys., Diffr., Theor. Gen. Crystallogr..

[cit68] Breiman L. (2001). Mach. Learn..

[cit69] Walters W. P., Murcko M. A. (2002). Adv. Drug Delivery Rev..

[cit70] O'Boyle N. M., Banck M., James C. A., Morley C., Vandermeersch T., Hutchison G. R. (2011). J. Cheminf..

[cit71] Anaconda Software Distribution, 2022, https://docs.anaconda.com/

[cit72] Raybaut P. , Spyder-documentation, 2009, Available online at: https://pythonhosted.org

[cit73] Oliphant T. E. , A guide to NumPy, Trelgol Publishing, USA, 2006, vol. 1

[cit74] McKinney W. , Python for high performance and scientific computing, 2011, 14, pp. 1–9

[cit75] Virtanen P., Gommers R., Oliphant T. E., Haberland M., Reddy T., Cournapeau D., Burovski E., Peterson P., Weckesser W., Bright J., van der Walt S. J., Brett M., Wilson J., Millman K. J., Mayorov N., Nelson A. R. J., Jones E., Kern R., Larson E., Carey C. J., Polat İ., Feng Y., Moore E. W., VanderPlas J., Laxalde D., Perktold J., Cimrman R., Henriksen I., Quintero E. A., Harris C. R., Archibald A. M., Ribeiro A. H., Pedregosa F., van Mulbregt P., SciPy 1.0 Contributors (2020). Nat. Methods.

[cit76] Hunter J. D. (2007). Comput. Sci. Eng..

[cit77] Tu C. , SALAM: an HTVS tool for organic materials, 2022, https://github.com/yidapa/salam

[cit78] Frisch M. J. , Trucks G. W., Schlegel H. B., Scuseria G. E., Robb M. A., Cheeseman J. R., Scalmani G., Barone V., Petersson G. A., Nakatsuji H., Li X., Caricato M., Marenich A. V., Bloino J., Janesko B. G., Gomperts R., Mennucci B., Hratchian H. P., Ortiz J. V., Izmaylov A. F., Sonnenberg J. L., Williams-Young D., Ding F., Lipparini F., Egidi F., Goings J., Peng B., Petrone A., Henderson T., Ranasinghe D., Zakrzewski V. G., Gao J., Rega N., Zheng G., Liang W., Hada M., Ehara M., Toyota K., Fukuda R., Hasegawa J., Ishida M., Nakajima T., Honda Y., Kitao O., Nakai H., Vreven T., Throssell K., Montgomery Jr J. A., Peralta J. E., Ogliaro F., Bearpark M. J., Heyd J. J., Brothers E. N., Kudin K. N., Staroverov V. N., Keith T. A., Kobayashi R., Normand J., Raghavachari K., Rendell A. P., Burant J. C., Iyengar S. S., Tomasi J., Cossi M., Millam J. M., Klene M., Adamo C., Cammi R., Ochterski J. W., Martin R. L., Morokuma K., Farkas O., Foresman J. B. and Fox D. J., Gaussian 16 Revision C.01, Gaussian Inc, 2019

