# Supplementary material for: Combining machine learning and quantum chemical calculations for high-throughput virtual screening of thermally activated delayed fluorescence molecular materials: the impact of selection strategy and structural mutations
Source: RSC Adv. 2022 Oct 31;12(48):30962–75. doi: 10.1039/d2ra05643g (PMC9619240; doi:10.1039/d2ra05643g)
Supplement: RA-012-D2RA05643G-s001 [file RA-012-D2RA05643G-s001.pdf]

Electronic Supplementary Material (ESI) for RSC Advances.
This journal is © The Royal Society of Chemistry 2022

Electronic Supporting Information for "Combining Machine Learning and Quantum Chemical Calculations for High-Throughput Virtual Screening of Thermally Activated Delayed Fluorescence Molecular Materials: the Impact of Selection Strategy and Structural Mutations"

Chunyun Tu,† Weijiang Huang,† Sheng Liang,‡ Kui Wang,† Qin Tian,† and Wei Yan*,†

†*School of Chemistry and Materials Engineering, Guiyang University, Guiyang, 550005, P. R. of China.*

‡*School of Mathematics and Information Science, Guiyang University, Guiyang, 550005, P. R. of China.*

E-mail: lrasyw@163.com

Phone: +86 1809 6050 905

Generation of initial compound library G0

The initial compound library G0 is constructed by combining donors with acceptors at preset connection sites (symbol * is used to denote the connection site). The structures of 30 donors is shown in Figure **S1**, and that of 43 acceptors is shown in Figure **S2**. After the enumeration of fragments, a random pick routine is used to further restrict the size to 1000.

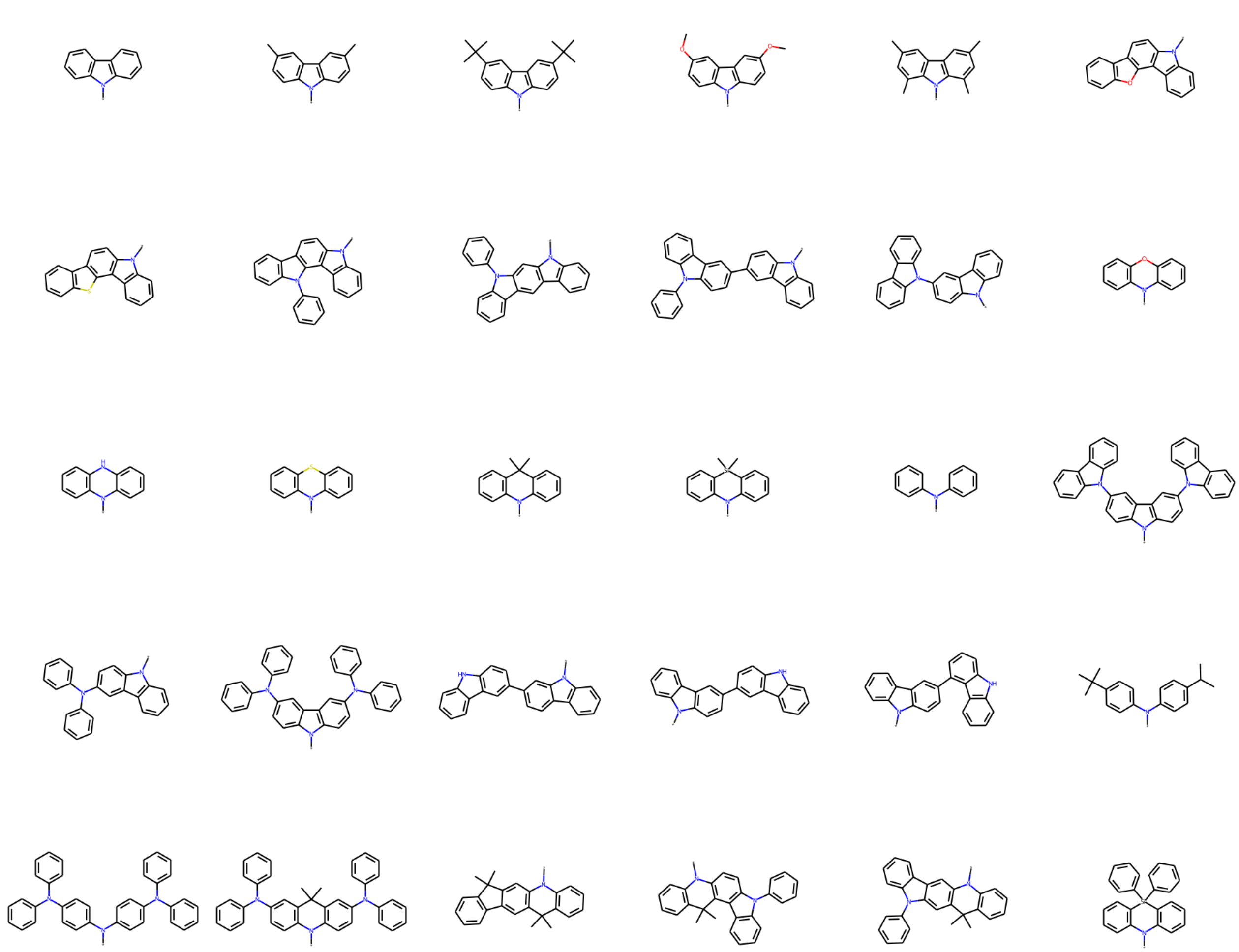

Figure S1: The donors (D) used as fragments for construction of donor-acceptor (DA) molecules (* is used to denote the connection site)

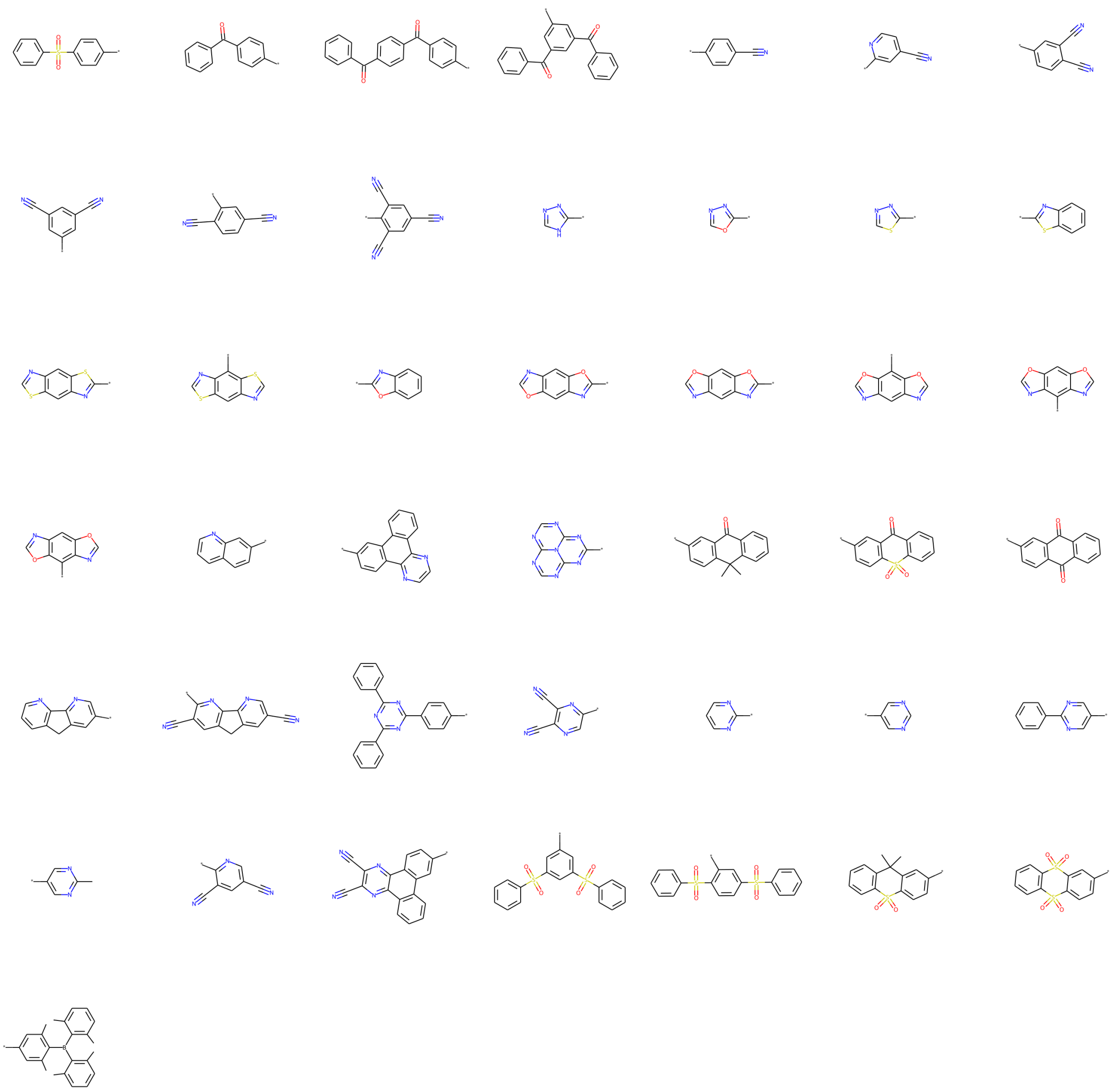

Figure S2: The acceptors (A) used as fragments for construction of donor-acceptor (DA) molecules (* is used to denote the connection site)

Effect of varied ground state geometry optimization methods

The difference in ground state geometries calculated by B3LYP/6-31G(d) and PM6-D3 levels of theory is measured by their root-mean-square deviations (RMSDs). The distribution of frequency of RMSDs is given in Figure **S3**. For a total of 72 molecules, only 25% of them have RMSD values larger than 0.69, hence, from a point of view of probablity, the replacement of B3LYP/6-31G(d) by PM6-D3 method seems acceptable.

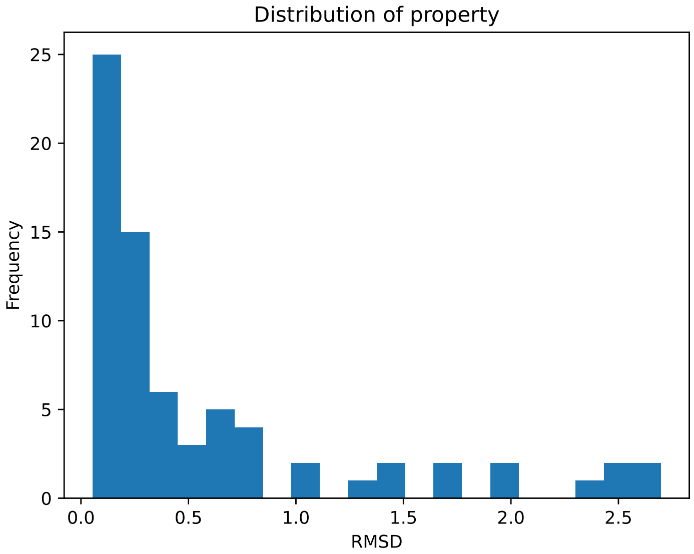

Figure S3: The distribution of frequency of RMSDs for **Sub3**.

The effect of varied ground state geometry optimization methods on the HTVS results have been briefly tested. The evolution of material abundance (ω_{MA}), average number of aromatic aCH bonds (n_{aCH}) and number of accumulated optimal molecules ($n_{acc\_opt\_mols}$) with increase of mutation generation (n_g) for **Sub3** using B3LYP/6-31G(d) level of theory as ground state geometry optimization method have been listed in Table **S1**. As compared with the semi-empirical method, the investment on a relatively high accuracy computational one seems to have the effect to speed up the convergence of ω_{MA}. Hence is beneficial for harvesting more optimal molecules at relatively low n_g.

Table S1: The evolution of material abundance (ω_{MA}), average number of aromatic aCH bonds (n_{aCH}) and number of accumulated optimal molecules ($n_{acc\_opt\_mols}$) with increase of mutation generation (n_g) for Sub3 using B3LYP/6-31G(d) level of theory as ground state geometry optimization method

n_g	ω_{MA}	n_{aCH}	$n_{acc\_opt\_mols}$
0	0.016	18.3	16
1	0.212	16.9	220
2	0.892	12.5	988
3	0.969	11.7	1808
4	0.960	11.8	2637
5	0.962	9.7	3482
6	1.000	8.0	4306

The evolution of skeleton (generic core) with mutation generation for Sub3

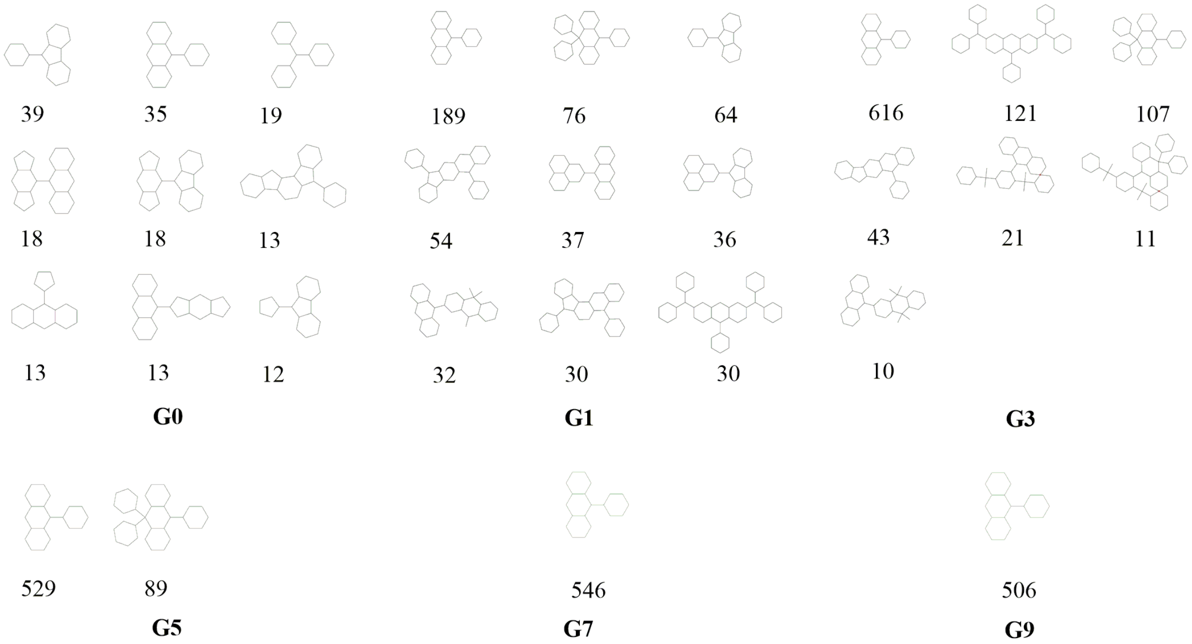

Figure S4: The evolution of skeleton (generic core) with mutation generation for **Sub3** (the numbers below structures denote the corresponding frequencies).

Details for training and evaluation of machine learning model

Table S2: The evolution of cross validation scores, mean and standard deviation of best ML models with increase of mutation generation (n_g) for Sub3

n_g	scores					mean	std
0	0.174	0.206	0.180	0.254	0.169	0.197	0.031
1	0.228	0.282	0.170	0.124	0.110	0.183	0.064
2	0.104	0.070	0.094	0.317	0.169	0.151	0.089
3	0.059	0.094	0.093	0.255	0.095	0.119	0.069
4	0.049	0.104	0.048	0.037	0.180	0.084	0.054
5	0.028	0.035	0.040	0.079	0.035	0.043	0.018
6	0.092	0.068	0.032	0.019	0.039	0.050	0.026
7	0.052	0.013	0.017	0.032	0.028	0.029	0.014
8	0.021	0.015	0.032	0.012	0.045	0.025	0.012
9	0.016	0.023	0.021	0.026	0.073	0.032	0.021

The featurization of structures of training molecules is carried out by utilizing the ECFP fingerprint (size = 2048) computing tool of the DeepChem package. By introducing the related tools of the open source machine learning Scikit-Learn package, a Random Forest Regressor (RandomForestRegressor, RFR) from the ensemble module is adopted as the ML model, Grid Search Cross Validation (GridSearchCV) and relevant score function and method (cross_val_score and neg_mean_squared_error) as tools for model selection, simple imputer (SimpleImputer) as data imputer, and a min max scaler (MinMaxScaler) for data scaling. The following grid parameters have been used for the Grid Search Cross Validation step: 'bootstrap': [True, False], 'n_estimators': [3, 10, 30, 100], 'criterion': ["mse", "mae"], 'max_depth': [2, 5, 10, 50], 'max_features': ["auto", "sqrt", "log2"]

The ECFP fingerprints as the X featurization vector, and the computed energy gaps (ΔE_{ST}) as the Y object vector. The X;Y is fed to the RFR model, by applying the GridSearchCV with above grid parameters, the cross_val_score method and 5-fold cross validation, the best ML model (best_reg) is screened out from the grid search hyper-parameter space. The best_reg is retrained with the training data, and is further evaluated by a 5-fold

cross validation using the same scoring method (neg_mean_squared_error). The newly learned ML model will be used for subsequent prediciting property of unseen molecules in the original compound library (n_g keeps unchanged).

To have a taste on the relative accuracy of the ML models, the related data for **Sub3** has been given in Table S2. Obviously, the mean and standard deviation are small enough, hence the ML model could be safely used to predict the energy gaps of unseen molecules with considerable confidence.

Optimal molecules sorted by SAS

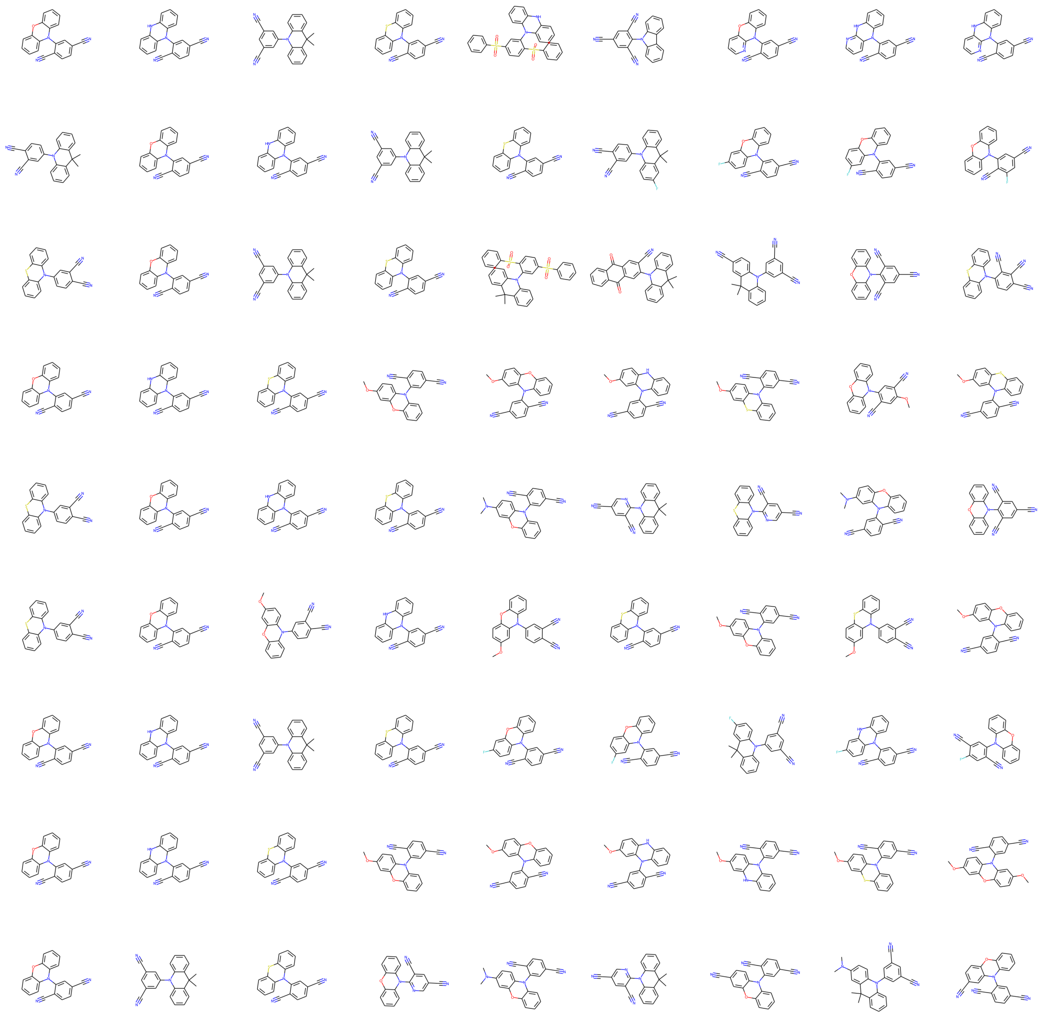

Figure S5: The structures of 9 optimal molecules with lowest SAS for all mutations except **Sub2** (each row corresponds to a mutation, row 1 $\rightarrow$ **Sub1**)

The (energy gap) optimal molecules for all mutations have been sorted by Synthetic Accessibility Scores from low to high, so as to give recommendation for TADF materials candidate. The structures of 9 molecules with lowest SAS for all mutations except **Sub2** have been depicted in Figure **S5**. Notably, duplication between different rows exists due to the origin setup of the computational road map.

Maximum Similarity Pairing Rule

The calculation of group fingerprint similarity (Δ_{MSPR}) is based on the following algorithm, which we name it the Maximum Similarity Pairing Rule (MSPR). Suppose we have two compound libraries L_A and L_B, which are represented by two SMILES-based string lists SML_A and SML_B.

(1.) Assume their sizes are m and n, and m $\leq$ n. If not, we exchange A and B.

(2.) Check whether there are intersection elements between SML_A and SML_B or not? If yes, the two libraries SML_A and SML_B can be represent by $SML_A = SML_{Intersection} + SML_{A1}$ and $SML_B = SML_{Intersection} + SML_{B1}$. Set the size of $SML_{Intersection}$ is p. Now, a mapping has been established between the intersection parts of the two libraries (one A molecule versus one B molecule).

(3.) Introducing ECFP fingerprint method to represent molecules and adopting Tanimoto metric, we can construct a (m - p) $\times$ (n - p) similarity matrix C whose matrix element c_{ij} is defined as the Tanimoto similarity between $SML_{A1}(i)$ and $SML_{B1}(j)$.

(4.) Find the larget element of the similarity matrix C, assume it is c_{kl}, a row exchange (k to 1) followed by column exchange (l to 1) would shift the larget element to the (1,1) position. At the same time, the exchange (k to 1) positions is applied on a (m - p) index list to trace the change of order of molecules in SML_{A1}. Similar manipulation applies to index list of SML_{B1}.

(5.) Find the larget element of the submatrix for the old C matrix, which is constructed by deleting the (1,1) element containing row and column. Shift that element to (2,2) position, and apply similar manipulation on index lists.

(6.) Repeat step (5) until the submatrix containing only 1 element.

(7.) Take out the main diagonal elements of matrix C as diagonal matrix D. The Δ_{MSPR} is calculated by Eq 1

$$\Delta_{MSPR} = \frac{p * 1.0 + \Sigma_i d_{ii}}{m} \tag{1}$$

By applying the algorithm, the two libraries succeed in building a mapping, which maps m molecules in A library with m molecules in B library (one versus one). Notably, the algorithm may not be numerical stable when degeneracy exists. Fortunately, for the cases we studied, it seems working well.

Optimal Generic Cores for Studied Mutations As $n_g = 9$

The optimal skeletons (generic cores) for studied mutations as $n_g = 9$ have been depicted in Figure **S6**.

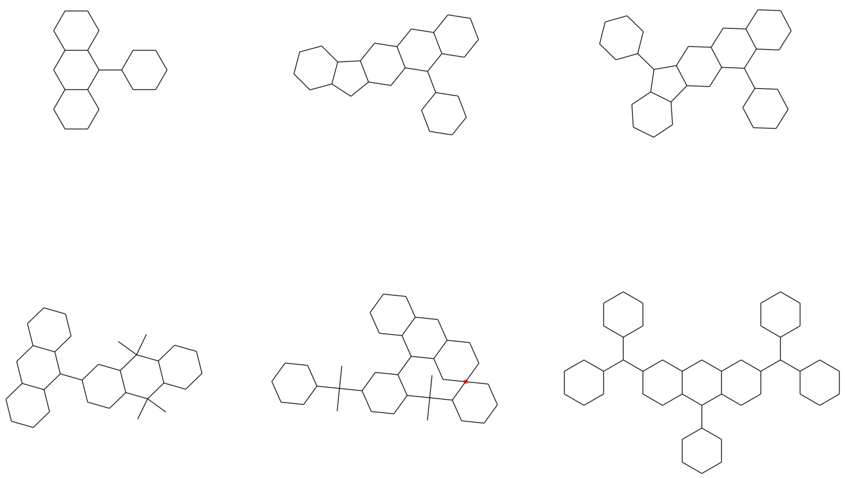

Figure S6: The optimal skeletons (generic cores) for studied mutations as $n_g = 9$
